# Supplementary material for: Study protocol for a phase III randomised controlled trial of Sailuotong (SLT) for vascular dementia and Alzheimer’s disease with cerebrovascular disease
Source: PLoS One. 2023 Mar 15;18(3):e0265285. doi: 10.1371/journal.pone.0265285 (PMC10016672; doi:10.1371/journal.pone.0265285)

# CLINICAL TRIAL PROTOCOL

A Multicentre, Randomised, Double-Blind, Placebo Controlled Trial to Evaluate the Effectiveness and Safety of Sailuotong (*SLT)*, a Standardised Chinese Herbal Medicine Formula in Patients with Vascular Dementia and Alzheimer's Disease with Cerebrovascular Disease

Short title: Clinical Trial of SLT for Vascular Dementia

Version 11


CHIEF INVESTIGATORS

Professor Dennis Chang, Western Sydney University, Australia (Coordinating Chief Investigator)

Professor Daniel Chan, Bankstown Hospital, University of New South Wales, Australia

Professor Henry Brodaty, University of New South Wales

Professor Alan Bensoussan, Western Sydney University, Australia

Dr Diana Karamacoska, Western Sydney University, Australia

Trial Protocol No: SLT001, Revised 16 December 2020

A Multicentre, Randomised, Double-Blind, Placebo Controlled Trial to Evaluate the Effectiveness and Safety of Sailuotong (*SLT)*, a Standardised Chinese Herbal Medicine Formula in Patients with Vascular Dementia and Alzheimer's Disease with Cerebrovascular Disease

**Co-ordinating Chief Investigator:**


Professor Dennis Chang
NICM Health Research Institute (NICM)
Western Sydney University
Bldg J, Western Sydney University,
Westmead Campus, 158-160 Hawkesbury Road, Westmead 2145
Tel: (+61 02) 9685 4725
Fax: (+61 02) 9685 4760
E-mail: d.chang@westernsydney.edu.au


**Sponsor:** Australia Shineway Technology Pty Ltd
Unit 2806, 591-597 George St, Sydney, Australia
Contact: Ms LU Yun (Iris)
Tel: +86 316 5759866
Fax: +86 316 5759866

**PROTOCOL SIGNATURE PAGE**

**CONFIDENTIAL**

**Protocol No.: SLT001 VERSION 11 Date: 16 Dec 2020**

**SPONSOR REPRESENTATIVE**
Australia Shineway Technology Pty Ltd

Mr Chen Zhong ________ ________________________ ________________
Name Signature Date

Title: Vice President, Shineway

**COORDINATING CHIEF INVESTIGATOR**

I agree to conduct this clinical study in accordance with the design and specific provisions of this protocol.

I understand that I may terminate or suspend enrolment of the study at any time if it becomes necessary to protect the best interests of the study participants.

I agree to conduct and supervise this investigation and to ensure that all associates, colleagues, and employees assisting in the conduct of this study are informed about their obligations in meeting these commitments.

I will conduct the study in accordance with Good Clinical Practice, the Declaration of Helsinki, and the moral, ethical and scientific principles that justify medical research. The study will be conducted in accordance with all relevant laws and regulations relating to clinical studies and the protection of patients.

I will ensure that the requirements relating to Human Research Ethics Committee (HREC) review and approval are met. I will provide Australia Shineway Technology Pty Ltd with any material which is provided to the HREC for Ethical approval.

I agree to maintain adequate and accurate records and to make those records available for audit and inspection in accordance with relevant regulatory requirements.

I agree to promptly report to the HREC any changes in the research activity and all unanticipated problems involving risks to human participants or others.

Dennis Chang ________________________ 16 DEC 2020 __________
Name Signature Date

Title: Professor, NICM, Western Sydney University

TABLE OF CONTENTS


1. INTRODUCTION
- 1.1 Background 5
- 1.2 Endpoints 6
- 1.3 Efficacy endpoints 6

2. LITERATURE REVIEW
- 2.1 Vascular Dementia 7
- 2.2 Complementary medicine for vascular dementia 8
- 2.3 Sailuotong (*SLT)*, a standardised herbal formulation for VaD 11

3. METHOD
- 3.1 Study design 15
- 3.2 Sample 16
- 3.3 Study protocol 20

4. ETHICAL CONSIDERATIONS
- 4.1 Ethical guidelines 29
- 4.2 Safety and tolerability of interventions 30
- 4.3 Implications for participants 31
- 4.4 Informed consent 32
- 4.5 Anonymity/ confidentiality/ privacy 33

5. ANALYSIS
- 5.1 Outcome measures 34
- 5.2 Statistical analysis 38

6. TIMEFRAME 40
7. REFERENCES 41

APPENDIX 1: Adverse Event Definitions 46
APPENDIX 2: Trial Contact Details 48
APPENDIX 3: Clinical Frailty Scale 54
APPENDIX 4: Contingency Plan (active during COVID-19 pandemic only) 55

# 1. INTRODUCTION

## 1.1 Background

Dementia is a leading cause of mental and physical disability in the elderly. Vascular dementia (VaD), which accounts for 15-20% of all dementia cases, is a syndrome of acquired cognitive functional impairment with a complex pathophysiological basis. Viable pharmaceutical options for VaD are currently lacking. The conventional VaD drug regimen has tended to focus on single therapeutic targets such as cholinesterase (ChE) or glutamate receptors and does not appear able to address the complex and multi-system nature of VaD.

Combination therapies using multiple herbs are common in Chinese medicine. These complex chemical mixtures are believed to be able to enhance therapeutic efficacy through synergistic and multi-target mechanisms and are ideal for disorders such as VaD that have multifactorial/multisystem pathophysiological components. Based on this theory, our team have employed conventional pharmaceutical techniques to develop a novel, standardised herbal formulation, Sailuotong (*SLT*) targeting VaD. Data from the preclinical studies have shown significant improvements in memory functions and in pathogenic biochemical parameters in various animal dementia models. Appropriate safety of *SLT* has been shown in the acute and chronic toxicity and herb-drug interaction studies. A pilot clinical trial of a 4-month *SLT* treatment has demonstrated a significant improvement in cognitive function in patients with VaD. A larger scale clinical trial with a longer intervention period and greater statistical power is needed to confirm the efficacy and safety of this novel formulation for VaD.

Building on these encouraging results, we propose to undertake a rigorous phase III clinical trial in 238 mild to moderate VaD patients to evaluate the clinical efficacy of *SLT* treatment over a 52-week period. Cognitive and functional improvement, global assessment of change, quality of life and safety will be assessed regularly over a period of the intervention (52 weeks) and follow-up (13 weeks).

This project is innovative and will provide vital efficacy and safety data for this novel standardised formulation. Success in this project may lead to a breakthrough intervention for VaD. The project will help build complementary medicine research capacity through national and international collaborations.

## 1.2 Objectives

In this multicentre, randomised, double-blind, placebo controlled trial, we propose to evaluate the clinical effectiveness of *SLT* for the symptomatic improvement of VaD in 238 patients with mild to moderate probable VaD or Alzheimer's disease with cerebrovascular disease (AD+CVD). Specifically, the objectives of the study are to:

- determine the efficacy of the herbal intervention on cognitive function, activities of daily living, and quality of life, and
- monitor the safety of *SLT* as a treatment for VaD or AD+CVD during 52 weeks

We hypothesise that a 52 week treatment of *SLT* as compared with placebo will be clinically effective and well tolerated in participants with VaD or AD+CVD. The design of this study follows the European Medicines Agency's Guidelines on Medicinal Products for the Treatment of Alzheimer's Disease and Other Dementia.[1]

## 1.3 Efficacy Endpoints

<u>Primary efficacy endpoints</u>

The primary efficacy endpoint will be the difference between *SLT* and the placebo groups in the changes in Vascular Dementia Assessment Scale-cognitive Subscale (VaDAS-cog) and Alzheimer's disease Cooperative Study-activities of daily living (ADCS-ADL) scores from Baseline and 26-, and 52-week follow-up visits.

<u>Secondary efficacy endpoints</u>

The secondary efficacy endpoints of the study will be the differences between the *SLT* and the placebo groups in the changes between baseline and 26-, and 52-week follow-up visits in:

- Scores of Clinician's Interview Based Impression of Change-plus (CIBIC-plus)
- Scores of additional executive function tests including CLOX and EXIT-25 from Baseline and 26-, and 52-week follow-up visits
- Scores of DEMQOL from Baseline and 26- and 52-week follow-up visits
- Scores of *Neuropsychiatric Inventory-Clinician rating scale* (NPI-C) from Baseline and 26- and 52-week follow-up visits
- Changes in MRI biomarkers from Baseline and the end of treatment assessment (52 week follow-up)

Safety endpoints

- Number of Adverse events and serious adverse events will be monitored during the course of the study.
- Changes in liver, renal, coagulation will be monitored during the course of the study.

# 2. Literature Review

## 2.1 Vascular Dementia

Dementia is a leading cause of mental and physical disability in the elderly and carries the highest disability weighting of all illnesses. VaD is a clinical syndrome of acquired intellectual and functional impairment that results from cerebrovascular and cardiovascular diseases and is the second most common cause of dementia after Alzheimer's disease (AD), accounting for 15-20% of all cases in western countries.[2] The prevalence of VaD is between 1 and 4% in individuals over the age of 65 years.[3] This figure is doubled every 5 to 10 years and for individuals over the age of 85, the prevalence of VaD surpasses that of AD. In Australia, there were around 245,400 dementia patients (~37,000 VaD patients) in 2009 and this figure is estimated to reach 1.13 million (~170,000 VaD patients) by 2050.[4] In Asia and some developing countries, the prevalence of VaD is even higher, equaling or exceeding that of AD.[2] VaD significantly impacts on life quality of patients and imposes a huge financial burden on the community and health care system. The total cost of dementia (including VaD) to the Australian health system was estimated at $6.6 billion in 2002, representing 1% GDP and it may exceed 3% of GDP by 2050.[5] 'Ageing well, ageing productively' is a current national research priority and acknowledges the significant burden dementia imposes to the community and health care system.

Cognitive impairment is the primary symptom of VaD. VaD can also cause disturbance of mood and behaviour and reduce quality of life. The pathophysiology of VaD is complex. It incorporates interactions between vascular aetiologies (cerebrovascular disorders and vascular factors), changes in the brain (infarcts, white matter lesions, atrophy) and host factors (age, education).[6] The final common aetiopathogenic pathway usually attributes to a hypoxic, hypoperfusive or occlusive process resulting in ischemic damage in various areas of the brain, with subsequent cognitive and memory function impairment.[3]

Currently, effective pharmaceutical interventions for VaD are lacking; standard treatment largely focuses on symptomatic management and prevention of additional brain damage via recognition and control of risk factors such as hypertension and atherosclerosis.[7] Several classes

of pharmaceutical agents are used for the symptomatic management of VaD, among which ChE inhibitors and glutamate receptor antagonists are suggested as producing the best clinical outcomes.[3] The most commonly used ChE inhibitors include donepezil, galantamine, rivastigmine and memantine. In two randomised, double-blind, placebo control trials, donepezil demonstrated significant improvement in cognitive function, clinical global impression and activities of daily living in patients with probable or possible mild to moderate VaD.[8] The latest Cochrane review of galantamine in VaD demonstrated contradictory results. One of the two studies included in the review showed that galantamine treatment did not produce significant improvement in ADAS-cog and CIBIC-plus in VaD, while the other study reported a significant improvement in ADAS-cog but not in CIBIC-plus.[9] Rivastigmine is a dual ChE inhibitor, targeting both major forms of ChE - acetylcholinesterase and butyrylcholinesterase. The most recent Cochrane review did not identify any suitable trials to be included in the analysis. Several smaller scale studies however, indicated some beneficial effect of rivastigmine in VaD. However, these studies suffered from either low patient numbers or did not compare rivastigmine to a placebo.[10] Memantine is a glutamate antagonist and is suggested to produce neuroprotective effects via blocking NMDA-type glutamate receptors. Two recent clinical trials of memantine in mild-to-moderate VaD demonstrated a small but statistically significant improvement in ADAS-cog. However, this result was not supported by a clinical impression of change measured by the CIBIC-Plus.[11] In summary, the existing evidence to support efficacy of pharmaceutical agents in VaD is relatively weak and inconsistent.[12] Moreover, the long-term benefits of these interventions in VaD have not been validated.[12] As a result, none of these agents are currently indicated for VaD under the Australian Pharmaceutical Benefit Scheme but are licensed for VaD in the UK.

## 2.2 Complementary Medicine for Vascular Dementia

In the absence of satisfactory pharmacological therapy, many patients and their carers turn to complementary medicine. The use of herbal medicine for the treatment of ageing-related disorders was documented in the literature more than 2000 years ago in ancient China where herbal remedies were used to boost memory function and increase longevity.[13] A literature review conducted by our team has revealed a large number of preclinical and clinical studies indicative of the potential benefits of herbal medicines in VaD and/or dementia.[13] Specific herbs of particular promise include *Ginkgo biloba*, *Panax ginseng*, *Bacopamonniera*, Huperzine A (an alkaloid extract isolated from a moss, *Huperziaserrata*), *Crocus sativus* and vinpocetin

(vinca alkaloid derived from the leaves of *Vinca minor*). In reviewing these herbs and their actions, we have identified three of particular interest (*Ginkgo biloba*, *Panax ginseng*, *Crocus sativus*), which led to our series of preliminary studies described below.

Ginkgo biloba: *Ginkgo biloba* leaf extract (ginkgo) has been widely used for management of ageing-related memory disorders. The principal constituents of ginkgo are flavonol glycosides (e.g., quercetin and kaempferol) and terpenoids (e.g. ginkgolide and bilobalide).[14] Data from preclinical studies suggest that ginkgo decreases oxygen radical discharge and proinflammatory functions of macrophages (antioxidant and anti-inflammatory), reduces corticosteroid production (antianxiety), increases glucose uptake and utilization and ATP production.[15] In addition, ginkgo appears to improve blood flow by increasing red blood cell deformability, decreasing red cell aggregation, inducing nitric oxide production and antagonising platelet activating factor receptor.[15]

In healthy young adults, ginkgo treatment has been shown to improve speed of processing, working memory, executive function and cognitive function.[16] A recent systematic review revealed that memory enhancement is the most robust acute effect of ginkgo in young adults.[17] In clinical trials with dementia patients (including VaD) however, the effectiveness of ginkgo for enhancing memory and cognitive function remains controversial. Numerous controlled clinical trials demonstrated various levels of improvement in memory loss, concentration, anxiety and other symptoms associated with dementia.[13] For example, a randomized, double-blind, placebo-controlled trial of 216 participants with AD or VaD showed significant improvements in the attention and memory function in the EGb761 (a standard ginkgo preparation) treated group after 24 weeks treatment.[18] Two more recent studies also demonstrated therapeutic benefits of ginkgo, especially in patients with neuropsychiatric features of dementia.[19,20] In contrast, several clinical trials with greater numbers and longer interventions reported no difference between ginkgo and placebo interventions in dementia treatments, which led to the conclusion in a recent Cochrane systematic review that the existing evidence to support the use of ginkgo remains inconsistent and unconvincing.[21,22] However, a newly published meta-analysis, that considered the baseline risk in the assessment of treatment effect, has shown 6 months of treatment with ginkgo is associated with a significant improvement of cognitive function in dementia patients.[23]

It is worth noting that almost all existing clinical trials used the standardised ginkgo preparation, EGb 761. Assessment of other ginkgo preparations that may have different chemical profiles of

active constituents, and preparations where ginkgo is used as part of a formulation (possibly having synergistic effects with other components – see below) is lacking.

*Panax ginseng* (ginseng) is widely used to treat VaD and other dementias. The principal bioactive components of ginseng are ginsenosides, which have been suggested to have antioxidant, anti-inflammatory and anti-apoptotic effects.[24] Several studies have reported that ginseng modestly improved thinking and learning capacity.[25,26] A 12 month randomized controlled trial evaluating a ginseng extract for the treatment of VaD in China, found that ginseng significantly improved memory function.[27] Studies conducted by Stough and Scholey found that ginseng also produces an EEG profile similar to ginkgo, which is consistent with its role as a cognition enhancer.[28] In a series of acute and chronic studies of in humans, the ginseng established robust enhancement of secondary and working memory, which may be related to the ability of ginseng to normalise blood glucose levels.[29,30]

*Crocus sativus* (saffron) is commonly used in traditional Chinese medicine as an antidepressant, antispasmodic, and anticatarrhal. Data from *in vivo* and *in vitro* studies demonstrated that saffron possesses neuroprotective properties. Saffron extract has shown to improve learning and memory function in ethanol-induced memory impairment in mice and to ameliorate cerebral ischemia induced oxidative damage in rat hippocampus.[31,32] Crocitin, the principal constituent of saffron and a strong antioxidant, is suggested to be largely responsible for saffron's protective effect on the central nervous system.[31]

Multisystem approach of Chinese herbal medicine: Combination therapy underpins the philosophy of Chinese herbal medicine, where patients are generally treated with multi-herbal formulations. There is preliminary evidence that complex chemical mixtures enhance therapeutic efficacy by facilitating synergistic action and/or ameliorating/preventing potential side effects.[33,34] Synergistic effects can occur in many ways, including for example, where constituents from herbal extracts interact with one another to improve their solubility and hence the bioavailability.[34] Furthermore, constituents of complex herbal extracts can affect different targets, which make them ideal therapies for disorders such as VaD, which have multifactorial/multisystem pathophysiological components.[35] Clinical studies in healthy volunteers demonstrate the cognitive effects of a ginkgo-ginseng combination far outweigh those of either extract alone, suggesting the possibilities of synergistic interactions between the extracts.[36,37] Of particular relevance to this study is that this effect was most marked on those tasks which are known to rely on effective cerebral blood supply.

## 2.3 Sailuotong (*SLT*), a standardised Herbal Formulation for VaD

Formulation background: The research team at Xiyuan Hospital has utilised the multi-system approach of Chinese herbal medicine to develop a three-herb formula (*SLT*) combining specific dosages of *Panax ginseng C A Mey* (ginseng), *Ginkgo biloba L* (ginkgo), and *Crocus sativus L* (saffron) for the management of VaD. *SLT* represents a new generation of herbal formulation where the chemical and pharmacological profiles have been clearly defined using modern chemistry, pharmacognosy and pharmacology techniques. To determine the optimal ratio of the three herb extracts in *SLT*, eight different combinations of ginkgo, ginseng and saffron extracts have been studied using orthogonal pharmacokinetic design in the right middle cerebral artery ligation induced ischemia model in rats and Morris water maze model in mice to evaluate their efficacy in improving the ischemic area of the brain and memory function. The data demonstrated that the herbal extracts in descending order of significance were ginseng, ginkgo and saffron. The optimal ratio of the three herbal extracts was 5:5:1 and the optimal dosage of *SLT* extract was 22 mg/kg in mice.[38] This figure was converted to a human dose equivalent through a surface area dosage conversion and the consideration of the approved human doses provided in the Pharmacopeia of the People's Republic of China.[38]

Pharmacological studies of *SLT*: A series of preclinical pharmacokinetic and pharmacodynamic studies were conducted on the three individual herbs and in combination. The data from these experiments demonstrate significant improvements in learning and memory functions, pathogenic biochemical parameters in blood and brain tissue, and antioxidant capacity in various experimental dementia models.[39-43]

In an *in vivo* study, *SLT* (11, 22, and 44 mg/kg per day over 15 days) was administered to dysmnesia models in mice induced by scopolamine, reserpine, chlorderazin, sodium nitrite, and alcohol, respectively. Compared with the control, the middle and high dose treatment of *SLT* markedly decreased the error numbers and prolonged the latencies of dysmnesia in all active groups.[39] In a chronic cerebral hypoperfusion model induced by bilateral common carotid artery ligation in rats, 8 weeks treatment of *SLT* (ig) significantly shortened the persistent time for finding the platform in Morris Water Maze.[40] Activity of cholinesterase was also significantly decreased ($p<0.05$) while the ACh level was markedly increased in the brain tissue ($p<0.05$). In addition, the activity of superoxide dismutase (SOD) was significantly enhanced ($p<0.05$).[40] The effects of *SLT* on ACh were also investigated in an amyloid β-protein induced dementia model in mice.[41] After 30 days treatment of *SLT* (15.5 and 31.0 mg/kg per day, ig), ACh levels in the

brain tissue increased by 18.56% and 19.97%, respectively, when compared with the model group (Figure 1). Both changes reached statistical significance (P<0.05). Similarly, in a PDAPP$^{v7171}$ transgenic dementia model in mice, *SLT* treatment at 31 and 62 mg/kg per day i.g. over 12 weeks significantly increased ACh in both treatment groups, while serotonin (5-HT) levels in the brain tissue decreased significantly in the high dose *SLT* group only.[42] The effects of crocin alone (the principal active component of *Crocus sativus*) on ischemia/reperfusion (I/R) injury were investigated using a global or bilateral common carotid artery occlusion (BCCAO) model in mice.[43] Transient global cerebral ischemia (20 min), followed by 24 h reperfusion, significantly increased the production of nitric oxide (NO) and malondialdehyde (MDA) in cortical microvascular homogenates and decreased the activities of superoxide dismutase (SOD) and glutathione peroxide (GSH-px). Pre-treatment with crocin at 20 mg/kg, on the other hand, resulted in a significant decrease in MDA content (Figure 2) and a significant elevation in total antioxidant capacity (increased SOD (*p*=0.000) and GSH-px (*p*=0.001) activities).[43]

**Figure 1 Effect of Sailuotong (SLT) on ACh in Aβ-treated rats (n=12, Mean±SD). $^{b}$ P<0.05 vs control group; $^{e}$ P<0.05 vs model group**

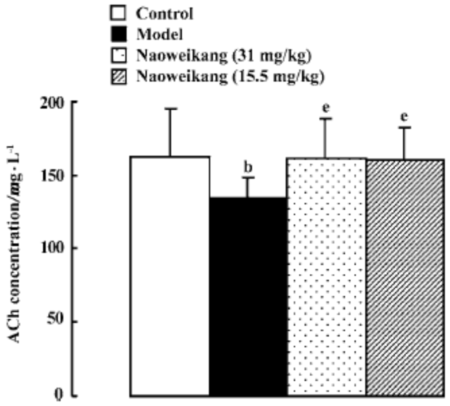


**Figure 2 Effect of crocin on MDA levels in cerebral microvascular homogenates after 20 min of BCCAO with 24 h reperfusion (n=10, Mean ± SD). ***p<0.001 compared with I/R group; ### p< 0.001 compared with I/R group with crocin treatment (5 mg/kg)**

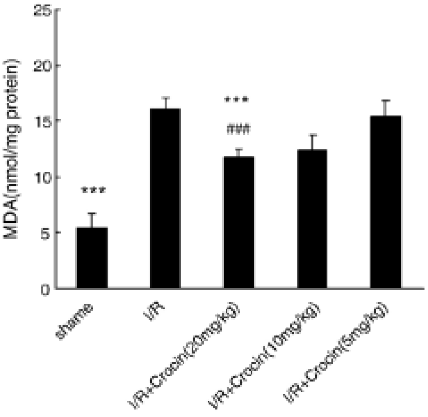


Animals studies in anesthetised dogs have also demonstrated that *SLT* treatment (8.25, 16.5 and 33 mg/kg over 24 h) significantly decreased the areas of focal cerebral ischemia/reperfusion injury in mice. A significant increase in cerebral blood flow was observed after administration of *SLT* (10 mg/kg for 60 - 180 min).[38] *SLT* treatment (16 mg/kg and 8 mg/kg for 7 days) also significantly decreased the platelet aggregation rate and whole blood viscosity in rats.[38]

Safety of *SLT*: Acute toxicity tests on *SLT* were undertaken in mice. The maximum tolerated dose (MTD) for the formula as whole, ginsenosides, flavone-glycosides and crocins was 1.7g/kg, 4 g/kg, 1.3 g/kg, and 5.0 g/kg, respectively, all of which were significantly higher than the proposed clinical dose (655x, 3422x, 1109x and 21386x respectively).[37] A long term toxicity study was also conducted in dogs. The MTD of *SLT* is 200 mg/kg, which is 70 times higher than the clinical dose.[37] In an *in vitro* study, the inhibitory capacity of *SLT* was evaluated on five cytochrome P450 isoenzymes recommended by the US FDA for drug interactions studies. The median inhibitory concentration (IC50) values for CYP1A2, CYP2B6, CYP2D6,

CYP2E1 and CYP3A4 were 50-140 fold higher than those potentially achievable by the therapeutic dose. This indicates that *SLT* treatment is unlikely to cause significant herb-drug interactions.[43]

Phase I studies: Two Phase I clinical studies were conducted including a single dose study and a multiple dose study. In the single dose study, 30 healthy volunteers were allocated to 7 *SLT* dosage groups receiving 60 mg, 120 mg, 180 mg, 240mg, 300 mg, 420 mg, or 540 mg of *SLT*, respectively. Each participant only received one dose starting from the smallest allocated dosage. A larger dosage would only be given when no adverse effect was reported in the previous dosage group. In the multiple dose study, 24 healthy participants were randomly allocated to the treatment and placebo control groups, each containing 12 participants receiving 180 mg or 300 mg of *SLT* Capsules and 3 or 5 placebo capsules over 14 days, respectively. The following adverse events were observed in the studies including stomach discomfort, urticaria, local skin pain, diarrhoea, itchy skin, dry mouth, heartburn, abdominal distension, dizziness, and nausea, among which dizziness, nausea, dry mouth, and abdominal distension were "unable to be determined" to relate to *SLT*. There is no significant difference in the proportion of these adverse events between the treatment group and placebo group in the multiple dose study. The distribution of adverse events was not dose-related. No abnormal *SLT*-related changes in liver & kidney functions and ECG were observed. In the single dose study, 86.7% (26/30) of the trial participants experienced various degrees of decline of the fibrinogen level but no dose-effect relationship was observed. In the multiple dose study however, no downward trends in fibrinogen level were found with the prolonged treatment of *SLT*. There was no significant difference in fibrinogen levels between the *SLT* and placebo groups and therefore the observed changes of fibrinogen in the single administration study may not relate to the trial medication.

Pilot trial of *SLT*: A pilot randomized, double-blind, placebo-controlled clinical trial of *SLT* was recently completed in Sydney with promising results.[44] Sixty-two patients (32 in the active group, 30 in the placebo group) with probable or possible VaD, were recruited according to the National Institute for Neurologic Disease and Stroke and the Association Internationale pour la Recherche et l'Enseignement en Neurosciences (NINDS-AIREN) criteria. Patients received 16 weeks of treatment with either active compound or identical placebo after randomization. At completion of treatment, the mean scores of the primary efficacy parameter, ADAS-cog reduced from 24.48 to 20.30 (mean reduction, 4.18± 0.75) in patients receiving *SLT* and from 18.98 to 17.81 (mean reduction, 1.18± 0.58) in patients receiving the placebo.[44] Although the baseline characteristics of ADAS-cog differed slightly (statistically insignificant) between the two

groups, ANCOVA analysis with the ADAS-cog baseline scores as a covariate showed that the improvement of ADAS-cog scores was significantly greater in the *SLT* group than those in the placebo group (p<0.005). These results are consistent with the findings from a Single Photon Emission Computed Tomography (SPECT) study of the brain, with a sub-group of patients (n = 7 *SLT*; n =11 placebo). The SPECT scan results showed that when compared to the placebo, the *SLT* treatment appeared to increase blood flow in the inferior frontal and anterior temporal lobes, and was more marked on the left.[44] These regions are known to be associated with cognitive, memory, auditory and speech functions of the brain. *SLT* also significantly reduced the degree of impairment in quality of life caused by VaD as evident by the significant improvement in SF36 scores.[44] Out of 8 domains, significant improvements were observed in the domains of 'role emotion', 'mental health', 'role physical' and 'social functioning' in patients receiving *SLT*, while significant trends towards improvement were also noted in two other domains - 'physical functioning' and 'bodily pain'. No significant differences were found in the placebo group. Two patients out of 64 recruited developed moderate insomnia and headache respectively within the first two weeks of the treatment. Both patients withdrew from the study and were later found to be in the *SLT* treatment group. In both patients, their symptoms subsided on discontinuation of the treatment. No serious adverse events were observed in this pilot trial.

# 3. METHOD

## 3.1 Study Design

This study will be conducted as a two-arm randomized, double-blind, placebo controlled clinical trial of 65 weeks, including a 52 week intervention and 13 week follow-up.

For a two-arm trial, participants will be randomized after informed consent is obtained, into two parallel treatment groups:

a. Intervention group taking *SLT* (Active)

b. Placebo control group (Placebo)

The trial will be double-blinded, such that the participants and investigators (including persons responsible for data collection, data management and data analysis) will not be aware of randomization assignments. No pilot study and no run-in period are required for this trial.

## 3.2 Sample

<u>Sample Size</u>

Following the release of the Phase II SLT trial results[46], sample size for this study was recalculated as the two studies used very similar participant cohorts and study design. In particular, the Phase II study provided useful data for VADAS-cog, one of the primary outcomes for the current study. Sample size was calculated using the Phase II trial outcome data for the two primary measures (VADAS-Cog and ADCS-ADLs). Based on their VADAS-Cog data[46], we expect the minimum between-group endpoint to be 3 or more units (with a standard deviation of 6.56) for a clinically meaningful change in cognition. Using a one-sided independent samples t-test for the purpose of calculating sample size, a total of 166 participants are required to detect this difference with 90% power at the 0.05 significance level. For the ADCS-ADLs, we anticipate the minimum between-group endpoint to be 3 or more units (with a standard deviation of 5.31) for a clinically meaningful change in daily functioning. Using a one-sided independent samples t-test for the purpose of calculating sample size with this measure, a total of 110 participants are required to detect this difference with 90% power at the 0.05 significance level.

Allowing for a total non-compliance/withdrawal rate of 30%, 238 participants will be recruited into the trial across all centres to ensure 166 participants in the final analyses.

For the MRI scanning sub study, to calculate the number of participants required repeated measures analysis of variance was used to examine the power of this study comprising 50 samples with two matched groups measured at two time points. Between factor repeated measures analysis was used to compute the power using the software G*power.[47] Assuming a medium effect size of 0.25, between-measures correlation of 0.5 at 5% level of significance the power was found to be 52%. However accounting for multiple hypotheses tests within the project the power may be reduced significantly. All participants who agree to participate in the main study will be asked if they would like to undergo MRI scanning until 50 participants are recruited. Although it is not possible to ensure equal numbers of treated and placebo participants undergo MRI scanning, that participants will be recruited into the main study in blocks of 6, each of which contain an equal number of treatment and placebo groups, it is likely that the number of treated and placebo participants in the MRI group will be similar.

Participant Characteristics

To achieve the aim of this study, it is desirable that the participant group is relatively homogenous with regard to health profile and disease risk factor presentation to ensure internal validity of the results. However, a broad representation of Australian adults according to demographic and social factors will be sought to improve generalisability of the study findings

for the community. The total number of participants with AD+CVD should not exceed a third of the sample size. Participants of each gender, a wide range of ages, ethnic and social backgrounds will be represented in this study.

*Inclusion Criteria*

To participate in this study, participants must be:

- 40-85 years old*;
- Outpatients diagnosed with either probable VaD as defined by the National Institute of Neurological Disorders and Stroke (NINDS) and the Association Internationale pour la Recherche et l'Enseignement en Neurosciences (AIREN) (Roman et al, 1993) or possible Alzheimer's Disease as defined by the National Institute of Neurological and Communication Disorders and Stroke (NINCDS)-Alzheimer's Disease and Related Disorders Association (ADRDA) (McKhann et al, 2004) with significant neuroimaging (CT or MRI) evidence of cerebrovascular disease;
- An MMSE score between 10 - 24 for the diagnosis of mild to moderate dementia;
- Absence of severe depression (Geriatric Depression Scale 15-item version, total score ≤ 11);
- Stable or controlled by optimal medication over a minimum of 6 months if on cholinesterase inhibitors and 3 months for (if present) hypertension, diabetes, cardiac disease or stroke, or if on hypnotics and sedatives, stabilised for more than 3 months prior to inclusion in the study;
- Agreement to take part in the study as evidenced by a personally signed and dated informed consent document indicating that the subject (or a legally acceptable representative if the subject is unable to provide consent), has been informed of all pertinent aspects of the study;
- Participants must also be accompanied by a study partner/carer, and this person must be able to assist the participant comply with the study protocol. This requires the study partner/carer to be in contact with the participant at least 2 days per week;
- If female, has no intention to become pregnant during the study.

* *Rule waiver:* a participant aged over 85 years may still be eligible for enrolment if he/she fulfils the following two conditions:

(1) the participant meets all inclusion and exclusion criteria of the study;

(2) participant's health status is likely to allow for successful completion of the 15-month study based on clinical judgement of the principal investigator after assessing the participant's medical history, current health and comorbidities and factors potentially affecting compliance (e.g., availability of a reliable study partner/carer). A 9-point Clinical Frailty Scale (see Appendix 3) will be used to facilitate PI's decision making process. Clinical Frailty Scale is a well validated tool used clinically to predict death or need for institutional care in elderly (Rockwood et al., CMAJ, 2005; 173:489-495). The participant may be considered suitable for this study if his/her frailty score is less or equal to 5 (mildly frail). In such a scenario, a rule waiver will be completed before enrolment.

*Exclusion Criteria*

Participants will be excluded from the trial if they:

- Have other types of dementia and/or severe form of delirium, depression, schizophrenia, acute illness or poorly controlled chronic diseases;
- Receive administration of the ingredients in the *SLT* formula (ginseng, ginkgo and saffron);
- Have a history of severe forms of peptic ulcers, diabetes with complications, pulmonary disorders, renal and/or hepatic disorders;
- Abnormal pathology test results: Cr > 1.5 times upper limit of normal (ULN); Alt, AST or ALP > 2 times ULN; PT > 3 second more than ULN; APTT > 10 seconds more than ULN; Plt < $100x10^9$/L;
- Have a stroke in the 3 months before screening;
- Have severe dysphasia, mental retardation and/or life expectancy < 6 months;
- Are allergic to more than 2 medications or at least 1 ingredient of *SLT*;
- Are participating in another clinical trial;
- Are pregnant or lactating.

*Additional Exclusion Criteria - MRI Sub-study*

- Allergy to Gadolinium contrast medium
- Pacemaker implanted;
- Have any wires, clips, or other metal implanted;
- Have a cochlear implant.

**Discontinuation of Participants from Treatment or Assessment**

Should any of the above exclusion criteria present after enrolment of the study, the Investigator will be required to immediately contact the Chief Investigator to determine the next appropriate course of action.

Participants who enter the trial, finish their baseline examination, are allocated to randomised treatment and begin taking the blinded medication will be counted as withdrawal cases if, during the study if they:

- Become pregnant;
- Withdraw informed consent. Participants are free to discontinue their participation in the trial at any time, without prejudice to further treatment;
- Become lost to follow-up during the 52-week trial period;
- Demonstrate significant protocol non-compliance as determined by the investigator; or
- The Investigator considers that it is not in their interest to continue the study.

The study may be discontinued at any time by the sponsor or the Coordinating Chief Investigator on the basis of new information regarding safety or efficacy. Additionally, the study may be terminated if progress is unsatisfactory. Withdrawn/discontinued participants will not be replaced.

**Procedures for discontinuation**

In case of premature termination or suspension of the trial, the coordinating chief investigator must inform the trial participants and ensure appropriate follow up and therapy. In addition, the regulatory authorities and ethics committee must be informed.

Participants may withdraw/discontinue from the study at will at any time without explanation. The participant may also be withdrawn at the discretion of the Investigator due to a safety concern or if judged non-compliant with trial procedures. Participants that withdraw/discontinue from the study will be asked to attend the end of treatment visit (Visit 8, Week 52) to monitor their safety, with a window period of up to 14 days of their withdrawal. The reason for withdrawal and date of withdrawal must be documented in the participant's source data file and electronic Case Report Form (CRF). All AEs and SAEs must be followed until resolution or stabilisation unless, in the Investigator's opinion, the condition is unlikely to resolve.

**Concomitant Treatment**

Participants may take routinely prescribed medications provided the relevant condition has been stable or controlled by optimal medication for more than 3 months except those drugs prohibited by the Exclusion Criteria. For other medications (including herbal medicine and nutritional supplements), which may have cognitive effects (e.g., cholinesterase inhibitors, beta blockers, angiotensin receptor blockers), the doses should be stable for at least 6 months for ChE inhibitors and 3 months for all other medications prior to the commencement of the trial and remain stable for the duration of the study. The details of all medical treatments and health management plans will be recorded as part of the screening process and then, following enrolment, during every follow-up visit. Notification of a person's enrolment in the trial and the implications for management will be communicated, with their consent, to their General Practitioner. It is anticipated that the presence or effect of any existing treatment confounders or effect modifiers will be equally distributed between groups due to the randomisation process.

## 3.3 Study Protocol

**Figure 3. Schematic Trial Protocol**

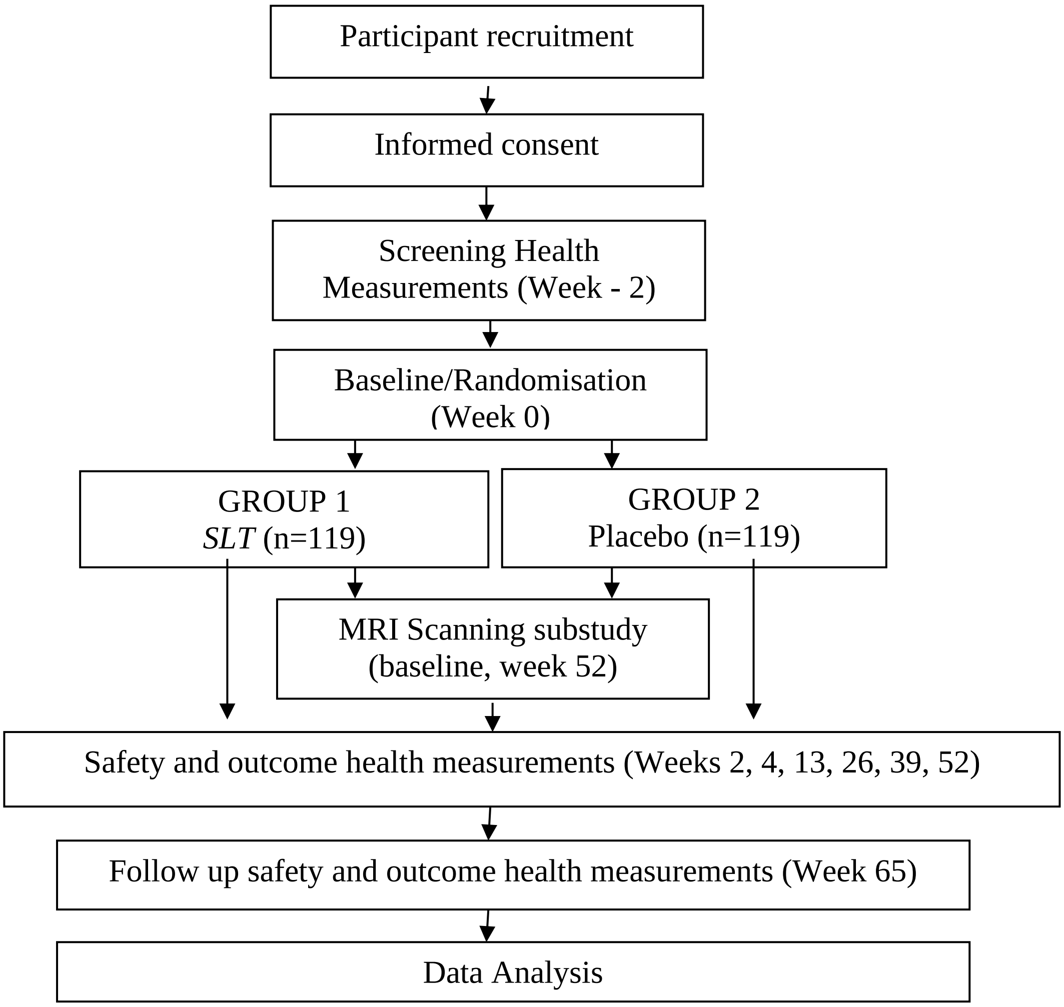


<u>Recruitment Protocol</u>

Recruitment strategies to be used in the Australian sites will include:

- All clinicians involved in this trial and their associated hospitals, clinics and networks
- Advertising through Alzheimer's Australia website
- Visits by the investigators to major aged care outpatient and nursing home facilities
- Visits to Division of General Practice meetings/events
- Posters and brochures in aged care, general practices and other health facilities
- Recruiting through Step Up For Dementia Research website
- Media resources of Western Sydney University e.g. advertisements in local newspapers
- Advertising through newsletters and websites of NICM Health Research Institute (NICM), Western Sydney University
- Advertising through online social media platforms (e.g., twitter, Facebook, Instagram)

Resources required for the recruitment phase of the trial include advertising materials, Health Practitioners Information Packages (information sheets for practitioners, information sheets for administration staff, information sheets and contact cards for potential participants). Participants may also be directly mailed information about the trial. Participants can take as much time as is required to consider their participation.

Screening Appointment Protocol

Based on our team's previous experience with recruitment rates for the VaD pilot trial, it is anticipated that 238 participants can be recruited over a 24 month period. The participants will be assessed and classified according to the NINDS-AIREN or NINCDS-ADRDA diagnostic criteria.[48] Mini-mental Status Examination (MMSE) will also be recorded to distinguish types and severity of dementia. A sub-set of 50 participants will undergo Magnetic Resonance Imaging (MRI) scans. Other assessments will include: history and examination, blood tests for reversible dementia, Computer Tomography (CT) scans, and neuropsychological assessment if deemed necessary.

Randomisation and Allocation Concealment Protocol

Randomisation will be conducted external to the primary research team (investigators listed at the start of this document) by a research officer of the National Institute of Complementary Medicine (NICM). This person will be responsible for producing computer generated randomisation treatment sequences which randomly associate a randomisation number with either active or placebo treatment. NICM's Clinical Trials Manager and Strategic Operations Manager are responsible for securely storing the randomisation allocation in a restricted access electronic file.

Randomisation numbers will be allocated in permuted blocks of 6 randomisation numbers starting from 001 to 238 with each block containing 3 active and 3 placebo assigned randomisation numbers. Sites will allocate the randomisation numbers in order of number sequence starting with the lowest number in each block and using all numbers in a block of 6 before starting with the next block of numbers. As soon as the randomisation number is assigned, it will be recorded on the screening log, in the participant source data file and electronic Case

Report Form (eCRF). Details of any participants randomised out of sequence will be notified immediately to the Coordinating chief investigator.

Investigators and/or pharmacists may request to break the blind for a participant if there is a clinical need to know the treatment allocation for a subject e.g. to manage the treatment of a serious adverse event. The site's Principal Investigator needs to discuss the reasons for breaking the blind with the Coordinating Investigator (or if unavailable, the Trial Coordinator). If this request is approved, it will be forwarded to the NICM Clinical Trials Manager. The NICM Senior Manager and Clinical Trials Manager will liaise with the Chief Investigator to process the code break request. When a participant allocation blinding is revealed, the participant ID, date, reason for opening, name of person and signature of who broke the seal, should be provided in writing. The Principal Investigator, Chief Investigator and Statistician will be notified of this information. The participants, principal investigators, research team members, trial medical practitioners, data collection nurses and other staff of the trial centres will have no knowledge of the randomisation assignments until follow-up outcome measures and data analysis are complete.

MRI Substudy Protocol

Structural MRI, MRA and dynamic contrast enhanced (gadolinium) perfusion MRI, are performed on a GE 3 Tesla 750W wide bore scanner and 32-channel Nova head coil at Macquarie Medical Imaging (MMI). The following protocols will be completed within an hour session. Our structural scan (T1-weighted and FLAIR) protocols and diffusion scan protocols were developed in line with the proposal from the Alzheimer's Disease Neuroimaging Initiative (ADNI). The supervising neuroradiologist is Dr John Magnuson at MMI. Standardised protocol: 3D T1-weighted contiguous coronal sections through whole brain (T1-TFE sequence and 3D acquisition) with voxel size 1 x 1 x 1 mm3; 3D T2-weighted FLAIR contiguous axial slices whole brain with voxel size 1 x 1 x 1 mm3. Then we will perform susceptibility weighted imaging (SWI) for examining cerebral microbleeds. High directional (61-gradient directions) diffusion weighted imaging (DWI) will then follow. We will then carry out angiogram (MRA) scan before taking gd-perfusion scan which is for generating cerebral blood flow and volumes. We will then do a post-gadolinium enhancement volumetric T1-weighted (this has better grey/white matter contrast than the pre-gd T1-weighted). We have all these T1 and T2 volumetrics (including pre- and post-gd T1s) for an accurate estimation of brain volumetric

changes in 12 months. Images will be de-identified, and then transferred to a secure workstation at Centre for Healthy Brain Ageing (CHeBA), UNSW. This protocol will remain the same for the 12-month follow-up scans.

Treatment Description

Shineway Pharmaceutical Co. Ltd. will be responsible for manufacture and container preparation of the two trial intervention agents. The preparations will be manufactured in China in accordance with Australian TGA (Therapeutic Goods Administration) GMP requirements and regulations.

GROUP 1

Active is *SLT capsule*, composition:

- Ginsenosides powder – 27.27 mg
- Ginkgo flavone glycosides–27.27 mg
- Crocins – 5.46 mg

Participants in Group 1 will take 2 *SLT* capsules (60 mg/capsule), twice daily for 52 weeks.

GROUP 2

Placebo capsules will be created by the same manufacturer (Shineway Pharmaceuticals) to have similar colour, taste, texture and weight and will have no constituents which provide therapeutic effects. Participants in Group 2 will take 2 placebo capsules, twice daily for 52 weeks.

Medication Container Labelling Instructions

Each capsule container must state:

- Name of sponsor
- Pharmaceutical dosage form
- Batch/code number to identify contents and packaging operation (may be encoded for blinding purposes)

- Directions for use - take orally 2 capsules in the morning and 2 capsules in the evening with meals
- Statement "for clinical trial use only"
- A trial reference code
- Storage conditions
- Use-by or expiry date
- Statement "keep out of reach of children"

  And any other items required to be consistent with TG048 (Therapeutic Goods Order TG048)

## Clinical Laboratory Parameters and Abnormal Laboratory Test Results

For participants recruited in the Australian centres, tests will be performed by a certified pathology laboratory. Hard copies of all results will be provided to the investigator and transferred electronically to the clinical database.

The following laboratory tests will be performed at Screening, Week 4, Week 13, Week 26, Week 39, Week 52/EOT and Week 65:

| | |
|---|---|
| Haematology | Haemoglobin (Hb) |
| | Haematocrit (Hct) |
| | Red Blood Cell Count (RBC) |
| | Platelets Count (Plt) |
| | White Blood Cell Count (including a five part differential in absolute) |
| Coagulation testing | |
| | Prothrombin time (PT) |
| | Activated Partial Thromboplastin Time (APTT) |
| | Fibrinogen (FIB) Level |
| Liver Function | Total Bilirubin (TBil) |
| | SGOT (AST) |
| | SGPT (ALT) |
| | Alkaline Phosphatase (ALP) |
| | Albumin (ALB) |
| | Total Protein (TP) |

| | |
|---|---|
| Renal Function | Blood urea nitrogen (BUN)<br>Creatinine (Cr) |
| Electrolytes | Sodium (Na)<br>Potassium (K)<br>Chloride (Cl) |

The results of all laboratory tests required by the protocol will be recorded in the participant's source data file and in the subject's CRF. All clinically important abnormal laboratory tests occurring during the study will be repeated at appropriate intervals until they return either to the subject's baseline (i.e. the level recorded at Screening) or to a level deemed acceptable by the investigator and the clinical monitor (or his/her designated representative), or until a diagnosis that explains them is made.

Treatment Protocol

An investigator's brochure will be provided to guide all clinical trial staff in protocol details.

Participants will take two capsules of active or placebo medications, orally, twice per day for 52 weeks. Medications will be dispensed at baseline and week 13, 26 and 39 visits. Participants will attend clinic visits at baseline (week 0), and at 4, 13, 26, 39 and 52 weeks for progress and/or adverse event reporting with their sites principal investigator and to collect the next stage supply of medication. Participants and investigating staff will be blinded to treatment allocation until the end of the trial when the data analyses are completed, and the coding is unlocked. Assessments conducted between weeks -2 (screening) and 0 (baseline) will become the baseline assessments for the study. During the course of the study, assessments will be conducted at weeks 2 (via phone), 4, 13, 26, 39, 52 and 65 (follow-up) weeks after the commencement of treatment (See Table 1). Follow-up assessment questionnaires (65 weeks) will be completed after the treatment period and any adverse events will be closely monitored and reported. All participants will receive 12-months trial medication at the end of the 65-week follow-up period based on compassionate grounds as the product has not yet been made available for purchase in Australia as was anticipated. Provision of the complementary medication will be subject to the principal investigator's judgement and approval by the coordinating chief investigator. Participant's general practitioner and usual specialist (if relevant) will be informed of the participant's medication prescription and safety will be closely monitored during this period via telephone calls every three months, and requesting participants to continue to keep their adverse

event diary. Participants will also be asked to undergo identical pathology tests that have been conducted throughout the trial.

Medication containers will be labelled according to the randomisation schedule at an external location and transferred to all participating centres where they will be kept in a secure storage room and maintained below 30 degrees Celsius. At each appointment, an allocation of medication will be dispensed to participants by hospital pharmacists/research personnel according to the pre-coded container labelling. Participants will be required to return the original containers and any unused medication for monitoring. Any unused medication will then be destroyed locally with approval of the coordinating chief investigator. All delivered and dispensed, unused and returned quantities will be recorded in a medication log.

MRI Scanning Substudy

MRI scans will be performed in 50 VaD or AD + CVD participants in accordance with a standardised scanning protocol. Structural MRI, MRA and dynamic contrast enhanced (gadolinium) perfusion MRI will be performed on a GE 3 Tesla 750W wide bore scanner and 32-channel Nova head coil at Macquarie Medical Imaging (MMI) within 60 mins. Our structural scan (T1-weighted and FLAIR) protocols and diffusion scan protocols have been developed in line with the proposal from the Alzheimer's Disease Neuroimaging Initiative (ADNI). Standardised protocol: 3D T1-weighted contiguous coronal sections through whole brain (T1-TFE sequence and 3D acquisition) with voxel size 1 x 1 x 1 mm3; 3D T2-weighted FLAIR contiguous axial slices whole brain with voxel size 1 x 1 x 1 mm3. Then we will perform susceptibility weighted imaging (SWI) for examining cerebral microbleeds. High directional (61-gradient directions) diffusion weighted imaging (DWI) will then follow. We will then carry out angiogram (MRA) scan before taking gadolinium-perfusion scan which is for generating cerebral blood flow and volumes. We will then do a post-gadolinium enhancement volumetric T1-weighted (this has better grey/white matter contrast than the pre-gd T1-weighted). We have all these T1 and T2 volumetrics (including pre- and post-gd T1s) for an accurate estimation of brain volumetric changes in 12 months. Images will be de-identified, and then transferred to a secure workstation at Centre for Healthy Brain Ageing (CHeBA), UNSW. This protocol will remain the same for 12-months follow-up scans.

**Study Procedures: Schedule of Assessments**

In response to the COVID-19 outbreak in Australia, study procedures have been amended to protect the safety and wellbeing of participants, their caregivers as well as the research team, and to minimise the impact of the outbreak on the SLT001 trial. Recruitment is to be paused until the trial centre's public health directive and the relevant governance body allow for recruitment to resume. All COVID-19-related strategies are detailed in Appendix 4 of this document. Once the COVID-19 outbreak ceases as per the Australian government's directive, Appendix 4 will cease to be used.

**Screening Visit (Week -2 to 0)**

Participants eligible for study recruitment, and their study partner/carer, will have the nature, purpose, and risks of the study explained to them by the investigator. Participants (or person responsible), and their study partner/carer, agreeing to participate in the study will sign the informed consent documents. A unique subject screening number will be issued at the time of consent. The screening visit should occur within 2 weeks prior to randomisation (week 0).

Procedures will be performed in the following order:

- Informed Consent
- Medical history, prior and concurrent medications, and demographics will be documented.
- Body weight and height will be measured.
- Physical examination.
- Blood pressure, pulse rate.
- Neurological Assessment including NINCDS-AIREN diagnostic criteria, NINCDS-ADRDA diagnostic criteria, MMSE, GDS, and check of inclusion and exclusion criteria.
- Blood samples taken and sent to central laboratory for haematology, coagulation, liver function, renal function and electrolytes
- Check concomitant medications and procedures, including any planned hospital admissions.

**Baseline visit (Week 0)**

Participants who meet all of the inclusion and none of the exclusion criteria will be scheduled to return to the site for their Baseline visit. At Baseline, the following will be performed:

- Check of inclusion and exclusion criteria.
- Check concomitant medications and procedures and note any changes in the CRF.
- Complete assessment scales VaDAS-cog, ADCS-ADL, CIBIC-plus, DEMQOL, NPI-C, CLOX and EXIT-25
- Complete MRI scans for substudy (50 participants only).

**Week 2 Telephone contact**

- Record all AE/SAEs in the CRF

**Week 4 Visit**

- Check concomitant medications and procedures and note any changes in the CRF.
- Vital signs (HR, BP, Ht, Wt) will be measured
- Haematology, coagulation and liver and renal function tests
- Record all AE/SAEs in the CRF
- Record compliance rates

**Week 13 Visit**

- Check concomitant medications and procedures and note any changes in the CRF.
- Vital signs (HR, BP, Ht, Wt) will be measured
- Haematology, coagulation and liver and renal function tests
- Record all AE/SAEs in the CRF
- Record compliance rates and capture ‘success of blind’ information

**Week 26 Visit**

- Vital signs (HR, BP, Ht, Wt) will be measured
- Haematology, coagulation and liver and renal function tests
- Record compliance rates and capture ‘success of blind’ information
- Complete assessment scales VaDAS-cog, ADCS-ADL, CIBIC-plus DEMQOL, NPI-C, CLOX and EXIT-25
- Check concomitant medications and procedures and note any changes in the CRF
- Record all AE/SAEs in the CRF

**Week 39 Visit**

- Vital signs (HR, BP, Ht, Wt) will be measured
- Check concomitant medications and procedures and note any changes in the CRF
- Haematology, coagulation and liver and renal function tests
- Record all AE/SAEs in the CRF
- Record compliance rates and capture ‘success of blind’ information

**Week 52 Visit**

- Vital signs (HR, BP, Ht, Wt) will be measured
- Haematology, coagulation and liver and renal function tests
- Complete assessment scales VaDAS-cog, ADCS-ADL, CIBIC-plus, DEMQOL, NPI-C, CLOX and EXIT-25
- Check concomitant medications and procedures and note any changes in the CRF
- Record all AE/SAEs in the CRF
- Record compliance rates and capture ‘success of blind’ information
- Complete MRI scans for substudy (50 participants only).

**Week 65 Visit**

- Complete assessment scales VaDAS-cog, ADCS-ADL, CIBIC-plus

- Complete subject summary section
- Check CRF for completion and sign Investigator Declaration

**MRI Scanning Procedures**

MRI scans will be performed in 50 VaD or AD + CVD participants in accordance with a standardised scanning protocol. Each MRI session will take 60 minutes. MRI acquisitions will be performed in the following sequential order: a) volumetric (3D) T1-weighted (7 minutes); b) volumetric T2-weighted (3 minutes); c) volumetric Fluid-attenuated inversion recovery (FLAIR) (3 minutes) and they all have a spatial resolution of 1×1×1 $mm^3$. Then the following will be carried out: d) susceptibility weighted imaging (SWI) (7 minutes), diffusion weighted imaging (DWI, 61 gradients; 15 minutes), Gadolinium contrast enhanced perfusion (Gd perfusion) (2 minutes); following this we will perform a post Gd volumetric T1-weighted scan (7 minutes).

**Drug Accountability**

On an ongoing basis all study drugs will be reconciled against delivery, use and returned medication documents. Study drugs which are not used may be destroyed in a suitable facility locally after drug accountability has been completed and with confirmation from the coordinating chief investigator. To be compliant with the treatment protocol study, drug dosing must have a compliance rate of at least 70% over the 52-week study period. Treatment compliance will be calculated by dividing the actual number of capsules taken with expected number of capsules to be taken for the treatment period and multiplying by 100. Self-reported measures of compliance will involve asking participants and their study partner/carer to complete a diary between visits. The researchers will also ask about their adherence to the prescribed treatment regime at the week 4, 13, 26, 39 and 52 visits.

<u>Data Management Protocol</u>

The following source data records will be utilised:

- Participant recruitment log
- Participant screening log

- Subject medication dispensing log
- Medication accountability log
- Informed consent documentation
- Clinic notes
- Expenditure records
- Participant case record forms for screening and data collection. These records will be dated and signed for correct coding, completeness, accuracy and legibility by the chief investigators.
- Adverse event report form

# 4. ETHICAL CONSIDERATIONS

## 4.1 Ethical guidelines

This research will be conducted in accordance with the International Ethical Guidelines for Biomedical Research Involving Human Subjects prepared by the Council for International Organisations of Medical Sciences (CIOMS) in collaboration with the World Health Organisation (WHO)[49], the Australian National Statement on Ethical Conduct in Research Involving Humans[50] and the International Conference on Harmonisation of Technical Requirements for Registration of Pharmaceuticals for Human Use and the ICH Guidelines for Good Clinical Practice (CPMP/ ICH/135/95).[51]

Ethics approval will be sought from Human Research Ethics Committees (HREC) for all participating research institutions. The study and investigators will be accountable to these committees by way of ongoing update reports and by providing full access to source data documents.

The trial details will also be submitted to the TGA via CTN (Clinical Trial Notification) arrangements, which will include submission of notification fee and documents of ethical (HREC), sponsor, principal investigators and trial site approval.

## 4.2 Safety and tolerability of interventions

Acute toxicity tests on *SLT* were undertaken in mice. The maximum tolerated dose (MTD) for the formula as whole, ginsenosides, flavone-glycosides and crocins was 1.7 g/kg, 4 g/kg, 1.3g/kg and 5.0 g/kg, respectively, all of which are significantly higher than the proposed clinical dose

(655x, 3422x, 1109x and 20486x respectively).[38] A long term toxicity study was also conducted in dogs. The MTD of *SLT* is 200 mg/kg, which is 70 times higher than the clinical dose.[37] In an *in vitro* study, the inhibitory capacity of *SLT* was evaluated on 5 cytochrome P450 isoenzymes recommended by the US FDA for drug interactions studies. The median inhibitory concentration (IC50) values for CYP1A2, CYP2B6, CYP2D6, CYP2E1 and CYP3A4 were 50-140-fold higher than those potentially achievable by the therapeutic doses. This indicates that *SLT* treatment is unlikely to cause significant herb-drug interactions.[52]

Information about adverse events will be collected by means of a standard question at each visit: "Have you had any health problems since the previous visit?" and liver and renal function and routine haematological tests will be conducted at baseline, 4, 13, 26, 39 and 52 weeks after the commencement of the treatment. In addition, participants can contact the principal investigators in the event of concerning health problems. The investigators will contact the research physicians and/or other members of the research team, who will review the dosage and continuation status in the trial, provide medical advice and/or communication with their General Practitioner or other health professionals and co-ordinate emergency medical attention as is appropriate and required. Only in the event of discontinuation in the trial will the randomisation assignment for that participant be made known. If an adverse event is present when the clinical trial is terminated, its course will be followed until the event resolves or the investigator deems it to be unrelated to the trial and appropriate case management is organised.

The definitions for adverse events are presented in Appendix 2. As with most herbal medicines, some mild adverse events might be experienced by some participants. Chinese medicine theory suggests such events may be part of the detoxification and body balancing process, although no evidence can confirm this.  Examples of mild adverse events which might be encountered in this study are herbal sensitivity or intolerance symptoms of sore throat, skin rash, vomiting, nausea, asthma, headache, dry mouth, dizziness and insomnia. Moderate to major adverse events might involve impairment of liver and kidney function, abnormal clinical findings of non-physiological variance or other biochemistry abnormalities. Although serious adverse events (SAEs) are not anticipated for this trial, SAE procedures will be followed in these circumstances.

All adverse events will be recorded as part of trial records or submitted as required to Human Research Ethics Committee(s), TGA and the Department of Health. The reporting of adverse events will be consistent with TGA guidelines which are in turn based on the Note for Guidance on Good Clinical Practice (CPMP/ICH/135/95) and Note for Guidance on Clinical Safety Data

Management: Definitions and Standards for Expedited Reporting (CPMP/ICH/377/95)[51] and according to any requirements of the Human Research Ethics Committee.

## 4.3 Implications for Participants

Each participant will be required to contribute time to visit the allocated trial centre on eight occasions over a 65-week period.

- Screening consultation
- Baseline consultation
- 2-week telephone review of adverse events
- 4-week review
- 13-week review
- 26-week review
- 39-week review
- 52-week review
- 65-week review

The first occasion will involve a comprehensive consultation and health check-up with the principal investigator and a medical practitioner responsible for trial screening (including establishment of the diagnosis). Participants who do not fully meet the inclusion criteria will not be included in the study and will be notified of this outcome and any abnormal findings/test results from the screening. Their family doctors may also be contacted if the nature of these findings warrants this.

Subsequent visits for review and medication collection will be briefer. On five occasions (not at 0 and 65-week review), the participants will be required to have a blood sample taken, which may involve brief discomfort but poses no significant health risks. Participants will need to refrain from caffeine and smoking on the morning before blood testing and avoid alcohol and exercise for 24 hours prior to testing.  Participants will be reimbursed up to $30 for travel expenses and/or the time spent at each visit. In accordance with the above-mentioned ethical guidelines, a placebo will be utilised in this trial because there is no currently effective alternative medication for treatment of VaD. In addition, participants will continue to take their current prescribed medication.

A subset of 50 participants will undergo MRI scans. MRI scanning does not cause any pain, and the magnetic fields are not known to produce tissue damage of any kind. Some participants may experience discomfort when in an enclosed space or may find the noise of the scanner during

testing loud. Earplugs will be provided to prevent problems with noise and participants are able to communication with the technician throughout the procedure. These participants will also be reimbursed for their travel expenses to the MRI centre.

Participants will be insured for their involvement in clinical research through Western Sydney University.

## 4.4 Informed Consent

The protocol of informed consent will be conducted according to guidelines and appropriate national regulations outlined in Section 4.1. Although colleagues may introduce the project to potential participants, the principal investigators must provide written information and verbally discuss the trial with each person.

Informed consent will involve detailed provision at the appropriate level of comprehension for each person, about the purpose, methods, demands, risks, inconveniences, discomforts, benefits and possible outcomes of the research. Each person must exercise voluntary choice to participate, without coercion or inducement and recognise their choice will not impact on their relationships with the research team or their ongoing health management, and be informed that they are free to withdraw from the trial at any time.

The principal investigator will determine the participant's capacity to consent at the screening visit via a discussion of the trial process, and their understanding of it. Both written and verbal explanations of the trial process will be given. For individuals unable to provide personally signed and dated informed consent, guardians/carers of participants with cognitive impairment will be approached to provide consent on the participant’s behalf. The person responsible for a participant must meet the legislation and guardianship regulation requirements applicable to the State or Territory in which the trial is being conducted. The person responsible will also be given written and verbal explanations of the trial process and asked to provide written informed consent.

During the screening visit, the investigator must advise the participant and their carer of the possibility that the participant’s capacity to consent or participate in research may vary or be lost entirely. This matter will be discussed so the researchers can determine what course of action is to be taken at such times, and whether a person responsible is to be appointed to provide consent on the participant’s behalf. It is important that the investigators agree to comply with the participant's wishes; unless these circumstances would prevent the investigator acting in the participant's best interest. The capacity to consent must also be considered at the end of the

study, where the participant is due to complete the full 65-week study and is seeking to continue with the post-trial intervention. The principal investigator will determine whether it is safe for the participant to be administered this medication, and whether they have the capacity to consent to the process. In cases where the participant cannot provide consent, the person responsible will be contacted to discuss the nature of this post-trial intervention and provided with the option to consent for this medication on the participant's behalf.

The participant (or person responsible where required), study partner/carer, and the principal investigator will sign two informed consent forms: one for the investigator and one for the participant to keep, in addition to their information sheet. The consent form will state the name and contact details for the principal investigator and study co-ordinators for each recruitment site, who are to be available 24 hours a day, if they have questions or concerns.

It is not envisaged that culturally sensitive issues other than those potentially encountered in the day-to-day running of a clinic would present. All personnel involved in the trial are experienced with conducting clinical practice and clinical research such that cultural needs and beliefs are recognised. If the consenting individual is unable to read the information provided, an impartial witness will be asked to observe the verbal discourse of the trial and witness the signing of the consent form.

### 4.5 Anonymity/ confidentiality/ privacy

In accordance with ethical guidelines, the anonymity, confidentiality and privacy of participants will be protected.

The electronic clinical trial management data system and all electronic data will be password protected. Hardcopy source data records will be kept in a locked cabinet in the office of the principal investigator at each recruitment site during data collection and at completion of the study. Western Sydney University will keep source or certified copies and electronic data for an indefinite period of time.

All publication material will refer to general trial results as aggregate data and no individual participant names or identifying information will be released.

Participants are welcome to view their personal data at any time during the trial.

## 5. ANALYSIS

### 5.1 Outcome Measures

The outcome measurements to be employed in this study are:

Primary

- *Cognitive Test*: The Vascular Dementia Assessment Scale cognitive subscale (VaDAS-cog) will be used as the primary outcome measure of cognitive function in this trial. VaDAS-cog is modified version of the Alzheimer's Disease Assessment Scale cognitive subscale (ADAS-cog), which is a sensitive psychometric scale for assessing the severity of cognitive impairment over time in dementia participants and covers four core symptoms of dementia: memory, orientation, language, and praxis.[53] VaDAS-cog comprises additional frontal lobe subtests covering attention, working memory, executive function and verbal fluency to reflect the unique pathological feature of VaD.[54]

- *Functional Test*: Alzheimer's Disease Co-operative Study Activities of Daily Living Inventory (ADCS-ADL) is a validated instrument for assessing the level of functional disability in dementia patients and is gaining popularity in clinical trials for dementia.[55] ADCS-ADL scale is largely comprised of basic activities (B-ADL) and instrumental activities (I-ADL). B-ADL are simple activities (eating, walking, toileting, bathing, etc), whereas I-ADL are more complex activities (using the telephone, preparing a beverage or meal, using household appliances etc).

Secondary

- *Global Assessment of Change*: Clinician's Interview Based Impression of Change-plus (CIBIC-plus) is a reliable tool to rate participant's condition by a clinician experienced in the management of patients with dementia (global assessment). CIBIC-plus is a semi-structured interview and include four categories for evaluation – general, mental/cognitive state, behaviour and activities of daily living. It is estimated to take 30-45 min to complete.[56]

- *Additional Executive Function Tests:* Small vessel disease that causes subcortical ischemic impairments, including lacunar infarcts and ischaemic white-matter lesions, is the most common type of vascular dementia in elderly people.[57] Executive dysfunction, together with slowness of mental processing, early motor disturbances, and behavioural disorders are the main symptoms of vascular dementia. In this study, two additional instruments are employed for the further assessment of the executive function including CLOX and EXIT-25. Both instruments have been validated in various dementia cohorts including vascular dementia.[57-60]

- CLOX: CLOX is a modified version of a clock drawing task and has been proven to be quick and sensitive tool for the assessment of executive function in elderly dementia patients.[57,58] It has also been used a rapid method for dementia screening as it shows strong correlations with other traditional cognitive measures.[56]
- EXIT-25: EXIT-25 was developed by the same group of researchers who developed CLOX with the aim to define the behavioural sequelae of executive cognitive dysfunction in dementia patients.[59,60] It contains a 25-item interview scored from 0-50 and takes 15 minutes to complete. EXIT-25 correlates well with other executive control function tests such as Wisconsin Card Sorting Test.[60]

- *Quality of Life Assessment:* DEMQOL is recognised as one of the best dementia-specific health-related quality of life (HRQOL) instruments for mild to moderate dementia (MMSE $\geq$ 10 is ideal for the target cohort in this study).[62] DEMQOL has both self- (28-item DEMQOL) and proxy- rating (31-item DEMQOL-Proxy) components providing different but complementary perspectives on quality of life in dementia. DEMQOL also shows comparable psychometric properties to the best available dementia-specific measures.
- *Neuropsychiatric Inventory-Clinician rating scale* (NPI-C) is a revised version of the Neuropsychiatric Inventory (NPI). The latter is a popular assessment tool for neuropsychiatric symptoms based on a structured interview with a caregiver and comprises 10 neuropsychiatric domains including delusions, hallucinations, depression/dysphoria, anxiety, agitation/aggression, euphoria, dis-inhibition, irritability/lability, apathy, aberrant motor activity and night-time behaviour disturbances.[64] The NPI-C includes additional items, and a clinician-rating methodology and has demonstrated inter-rater reliability and convergent validity in dementia participants.
- *MRI Neuroimaging Assessment:* Neuroimaging MRI has increasingly been used in the study of healthy and impaired human cognitive functions associated with neurodegenerative disorders. A significant correlation has been shown between MRI activity and ADAS-cog performance in patients with early Alzheimer's disease.[63] Participants who agree to undergo MRI scanning will be asked to attend two MRI visits to investigate brain changes pre- and post-treatment/placebo (i.e. baseline and 12 months). Each test lasts approximately one hour. The following outcome measures will be assessed in the study:
    - A) volumetric changes of both treatment and placebo groups in 12 months by using 3D T1- and T2-weighted MRI scans (including pre- and post-Gd T1 scans). Both T1-

weighted and T2-weighted scans will be used as inputs for a multiple-channel segmentation algorithm for an increased accuracy of the computation. Post-Gd T1-weighted scans have better grey/white matter contrast and using both scans acquired in the same session will further improve the robustness and accuracy of volumetric estimation.

- B) Changes of white matter lesions by examining the volumetric changes of white matter hyperintensity (WMH) by using our own WMH pipeline.[66-67]
- C) Cerebral microbleeds and progress using SWI.
- D) Detailed white matter mapping and change of network properties using DWI scans and diffusion tensor imaging (DTI) analysis using DWI scans will be conducted.
- E) Accurate cerebral blood flow (CBF) and volume (CBV) will be calculated using Gd perfusion scans and to investigate changes of CBF/CBV of treatment and placebo participants. CBF measures how quickly is blood flowing through tissue, in units of mL/100g/min and CBV is the concentration of blood in a tissue, classically measured in units of mL/100g. Perfusion weighted Imaging Tools[69] will be performed to process Gd perfusion scans.

Analysis: Seven grey matter volumetrics will be taken into consideration in the 2 time-point data analyses, i.e. total brain grey matter volume, volumes of 4 cortical lobes, hippocampal volume and total subcortical volume. Three WMH measures will be included, i.e. total brain WMH, periventricular WMH and deep WMH volumes. One cerebral microbleeds measure, i.e. the count of microbleeds seen in the brain will be included. Further examination of 10 Gd perfusion variables, including 5 CBF (and CBV), i.e. CBF (and CBV) measures of 4 lobes and CBF (CBV) of hippocampus will be conducted.

<u>Safety Measures</u>

- *Liver and renal function, and routine haematological and coagulation (PT, APPT and fibrinogen) tests* will be conducted at baseline, 4, 13, 26, 39 and 52 weeks after the commencement of the treatment.

- All adverse events will be recorded at each visit, including those suspected to be related to the treatment (such as headache, dizziness, vomiting, allergy, etc) and any worsening of symptoms will be closely monitored. An assessment of the relationship between the adverse events or abnormal test results and the treatment will be made by the chief investigators and

trial physicians. An adverse event will be followed up until it resolves or for up to a month after the study is concluded. Serious adverse events (SAEs) will be immediately reported to the Human Research Ethics Committee granting approval and Suspected Unexpected Serious Adverse Reactions (SUSARs) to the TGA, in accordance with their guidelines. The causality of SAEs will be thoroughly investigated.

| Week | *-2 to 0* | *0* | *2* | *4* | *13* | *26* | *39* | *52* | *65* |
|---|---|---|---|---|---|---|---|---|---|
| | *Screening* | *Baseline* | | | | | | *End Tx* | *Follow up* |
| Window | | *0* | ±3 | ±7 | ±7 | ±7 | ±7 | ±7 | ±7 |
| Initial neurological assessment | X | | | | | | | | |
| Medical History | X | | | | | | | | |
| Vital signs (HR, BP, Ht, Wt) | X | | | X | X | X | X | X | |
| Physical Exam | X | | | | | | | | |
| NINDS-AIREN diagnosis | X | | | | | | | | |
| MMSE | X | | | | | | | | |
| GDS | X | | | | | | | | |
| Inclusion/Exclusion criteria | X | X - checked before randomisation | | | | | | | |
| VaDAS-cog | | X | | | | X | | X | X |
| ADCS-ADL | | X | | | | X | | X | X |
| CIBIC-plus | | X | | | | X | | X | X |
| NPI-C | | | | | | X | | X | |
| CLOX | | X | | | | X | | X | |
| EXIT-25 | | X | | | | X | | X | |
| DEMQOL | | X | | | | X | | X | |
| Haematology, coagulation, liver and renal function tests | X | | | X | X | X | X | X | |
| MRI scanning (if applicable) | | X | | | | | | X | |
| Adverse Events | | | X | X | X | X | X | X | |
| Con meds and procedures | X | X | | X | X | X | X | X | |

| | | | | | | | | | |
|---|---|---|---|---|---|---|---|---|---|
| Compliance, success of blinding | | | | | X | X | X | X | |

Table 1. Data collection timeline

## 5.2 Statistical Analysis

**Safety analyses**

There will be no interim analyses with the exception of monitoring safety variables. The frequency, type and probable association of adverse events will be tallied by de-identified treatment group every 3 months for review by the study team. If there is any significant safety issue identified an additional DMC meeting will be called.

**Preliminary analyses and data cleaning**

At completion of data collection, all variables and all logical pairs of variables will be subject to descriptive analyses using graphs, frequency counts and summary statistics. This will allow a) identification of unusual or unexpected results for data checking and b) familiarisation with the distributions and associations within the data set. Outcome variables which have severely non-symmetric distributions will be either transformed or categorised.

At the completion of data checking and correction, the data set will be locked for analysis.

**Checking for homogeneity of study centres**

As this is a multi-centre study, early analyses will address the question of whether or not there is heterogeneity between centres. Linear models will be fitted to each outcome measure in turn, with centre and centre by treatment added as fixed effects. Any statistically significant differences will be documented and explored further for potential confounding with demographic or medical history factors. If variation between centres cannot be explained (or is shown to be related to differences in study methods), the primary analysis will continue as planned, but will be followed by sensitivity analyses which will either stratify by or exclude the outlier site(s) (depending on the sample size of the site(s) involved).

**Demographic and baseline characteristics**

The demographic and medical characteristics of participants in each treatment group will be summarised using percentages or means and standard deviations. Pearson's Chi-square and independent samples t-tests will be used to check for any statistically significant differences

between groups. Results will be documented as p-values and, where necessary, addressed within the interpretation of study results.

**Missing data and protocol violations**

In the case of death, all measurements prior to death will be included in the analysis but all after death will be set to missing. To address withdrawals, loss to follow-up or non-compliance with the study analyses will be conducted on both an intention to treat (ITT) and per protocol (PP) basis. Withdrawing and non-compliant participants shall be encouraged to continue with data collection even if stopping treatment. Where data items are missing the last value carried forward method to replace missing data in the ITT analysis will be used. Participants who have significant deviations from the protocol will be removed from the PP analysis after the completion of the ITT analysis. Such significant deviations from the protocol will be determined and documented by the study clinician during the course of the study. Any deviation from randomisation, missing data and withdrawals will be fully reported for this purpose.

**Primary efficacy analysis**

The primary analysis will be linear mixed models through which we will test for differences between treatment groups on each outcome over time, with adjustment for random variation between treatment centres, with and without adjustment for other potentially important predicts (e.g. compliance, age, gender, severity of VaD). Non-linear changes over time will be tested by a) fitting time as categorical variable and b) testing for quadratic and cubic effects. Results will be reported as regression coefficients (or odds ratios if categorical) and associated 95% confidence intervals.

**Subgroup analyses**

Secondary analyses will be a repeat of the above stratified by disease type and, if necessary, with stratification by research centre.

Data will be analysed using SAS and/or SPSS software.

# 6. TIMEFRAME

| Month | 1-6 | 7-12 | 13-18 | 19-24 | 25-30 | 31-36 | 37-42 | 43-48 |
|---|---|---|---|---|---|---|---|---|
| Actives and placebo preparation | ■ | | | | | | | |
| Engagement of staff, final preparation, ethics approval | ■ | | | | | | | |
| Recruitment | | ■ | ■ | ■ | ■ | | | |

| | | | | | | | | |
|---|---|---|---|---|---|---|---|---|
| Intervention and follow-up | | | | | | | | |
| Data entry and analysis | | | | | | | | |
| Publications, Final Report | | | | | | | | |

Table 2. Research Timeline

# 7.REFERENCES


1. European Medicines Agency. Guidelines on medicinal products for the treatment of Alzheimer's disease and other dementia. London, 24 July 2008. Doc. Ref. CPMP/EWP/553/95 Rev. 1
2. Ogata J. Vascular dementia: the role of cerebral infarcts. [Review] *Alz Dis Assoc Dis*. 1999; 13:S38-48.
3. Aggarwal NT et al. Vascular dementia: emerging trends. *Seminars in Neurology*. 2007; 27:66-77.
4. Access Economics. *Keeping dementia front of mind: incidence and prevalence,* Canberra 2009.
5. Access Economics for Alzheimer's Australia. *The dementia epidemic: economic impact and positive solutions for Australia*. Canberra 2003.
6. Skoog I. Status of risk factors for vascular dementia. *Neuroepidemiology*. 1998; 17: 2-9.
7. Sachdev P et al. Vascular dementia: diagnosis, management and possible prevention. *MJA* 1999; 170:81-85.
8. Malouf R et al. Donepezil for vascular cognitive impairment. *Cochrane DB Syst Rev* 2004, Iss 1
9. Craig D et al. Galantamine for vascular cognitive impairment. *Cochrane DB Syst Rev* 2006, Iss 1
10. Craig D et al. Rivastigmine for vascular cognitive impairment. *Cochrane DB Syst Rev* 2004, Iss 2
11. McShane R et al. Memantine for dementia. *Cochrane DB Syst Rev* 2006, Iss 2
12. Moretti R et al. New treatment options for vascular dementia. Aging Health. 2007; 3:209-222.
13. Liu JG, Chang D. Vascular Dementia. *Journal of Complementary Medicine*. 2006; 5:14-20.
14. Smith JV et al. Studies on molecular mechanisms of *Ginkgo biloba* extract. *ApplMicrobiolBiotechnol*. 2004; 64:465-472.
15. Chan P-C et al. *Ginkgo biloba* leave extract: biological, medicinal and toxicological effects. *J Environ Sci Health Part C*. 2007; 25:211-244.
16. Scholey AB et al. Acute, dose-dependent cognitive effects of *Ginkgo biloba*, *Panax Ginseng* and their combination in healthy young volunteers: differential interactions with cognitive demand. *Hum Psychopharm - Clin and Exper*, 2002; 17:35-44.
17. Kennedy DO, Scholey AB et al. Modulation of cognitive performance following single doses of 120 mg *Ginkgo biloba* extract administered to healthy young volunteers. *Hum Psychopharm - Clin and Exper*2007;22**:**559–566.
18. Kanowski S et al. Proof of efficacy of the *Ginkgo biloba* special extract EGB 761 in outpatients suffering from mild to moderate primary degenerative dementia of the Alzheimer type or multi-infarct dementia. *Pharmacopsychiatry*. 1996; 29:47-56.

19. NapryeyenkoA et al. *Ginkgo biloba* Extract EGb761 in the treatment of dementia with neuropsychiatric features: A randomised, placebo-controlled, double-blind trail. 21st *International Conference of Alzheimer's Disease International*, Istanbul, 2005.

20. Mazza M et al. *Ginkgo biloba*and donepezil: a comparison in the treatment of Alzheimer's dementia in a randomised placebo-controlled double-blind study. *European Journal of Neurology*. 2006; 13:981-985.

21. Schneider LS. *Ginkgo biloba* extract and preventing Alzheimer's disease. *JAMA*. 2008; 300:2306-2308.

22. Birks J et al. Ginkgo biloba for cognitive impairment and dementia. *Cochrane DB Syst Rev* 2009, Iss1

23. Wang BS et al. Effectiveness of standardized Ginkgo biloba extract on cognitive symptoms of dementia with a six-month treatment: a bivariate random effect meta-analysis. *Pharmacopsychiatry*. 2010; Epub ahead of print.

24. Radad K et al. Use of ginseng in medicine with emphasis on neurodegenerative disorders. *Journal of Pharmacological Science*. 2006; 100:175-186.

25. Lun X et al. Observation on efficacy of CT positioning scalp circum-nEueedling combined with Chinese herbal medicine in treating poly-infarctional vascular dementia. *ZhongguoZhong Xi Yi Jie He ZaZhi* 2003; 23(6): 423-25.

26. Sorenson H, Sonne J. A double-masked study of the effects of ginseng on cognitive function. *CurrTher Res* 1996; 57(12): 159-68.

27. Jinzhou T et al. Ginseng may improve memory in stroke dementia patients. *American Stroke Association Meeting Report*. 2003

28. Kennedy DO, Scholey AB, et al. Electroencephalograph (EEG) effects of single doses of Ginkgo biloba and Panax ginseng in healthy young volunteers. *Pharm, Biochem Be* 2003; **75:**701–709.

29. Reay JL, Scholey AB, et al. *Panax ginseng* has no effect on indices of glucose regulation following acute or chronic ingestion in healthy volunteers. *Br J Nutr* In press.

30. Reay JL, Scholey AB et al. Effects of *Panax ginseng*, consumed with and without glucose, on blood glucose levels and cognitive performance during sustained 'mentally demanding' tasks. *J Psychopharmcol* 2006; 20:771-781.

31. Abe K and Saito H. Effects of saffron extract and its constituent crocin on learning behaviour and long-term potentiation. *Phytother. Res*, 2000; 14:149-152.

32. Hosseinzadeh H et al. Safranal, a constituent Crocus sativus, attenuated cerebral ischemia induced oxidative damage in rat hippocampus. *J Pharm Pharmaceut Sci*. 2005; 8:394-399.

33. Kroll U et al. Pharmaceutical prerequisites for a multi-target therapy. *Phytomedicine*. 2006;13;12-19

34. Wagner H et al. Synergy research: approaching a new generation of phytopharmaceuticals. *Phytomedicine*. 2009; 16:97-110.

35. Jia W et al. The rediscovery of ancient Chinese herbal formulas. *Phytother Res*. 2004; 18:681-686.

36. Scholey A and Kennedy DO. Acute, dose-dependent cognitive effects of *Ginkgo biloba, Panax ginseng* and their combination in healthy young volunteers: differential interactions

with cognitive demand. *Human Psychopharmacology: Clinical and Experimental*. 2002; 17:35-44.

37. Kennedy DO et al. Differential, dose dependent changes in cognitive performance following acute administration of a *Ginkgo biloba/Panax ginseng* combination to healthy young volunteers. *Nutr. Neurosci*. 2001;4:399-412.
38. Liu JX et al. 2008 *SLT for VaD*. Beijing:China Academy of Chinese Medical Sciences.
39. Xu L, Liu JX et al. Effect of Weinaokang (*SLT*) on dysmnesia in mice model. *Journal of Pharmacological and Clinical Chinese Herbal Medicine*. 2007; 23:60-61.
40. Xu L, Liu JX, et al. Effects of Weinaokang (*SLT*) capsule in tracephalic cholinergic system and capability of scavenging free radicals in chronic cerebral hypoperfusion rats. *China Journal of Chinese MateriaMedica*. 2008; 33:531-534.
41. Liu JX, et al. Effect of combination of extracts of ginseng and ginkgo biloba (*SLT*) on acetylcholine in amyloid beta-protein treated rats determined by an improved HPLC. *ActaPharmacologicaSinica*. 2004; 25:1118-1123.
42. Cong WH, Liu JX, et al. Effects of extracts of ginseng and ginkgo biloba on hippocampal acetylcholine and monoamines in PDAPPV7171 transgenic mice. *Chinese Journal of Integrated Medicine*. 2007; 27:810-813.
43. Zheng YQ, Liu JX, et al. Effects of crocin on reperfusion-induced oxidative/nitrative injury to cerebral microvessels after global cerebral ischemia. *Brain Research*. 2006; 1138:86-94.
44. Liu J, Chang D, Chan D, Liu JX, Bensoussan A. A Randomised Placebo-Controlled Clinical Trial of a Chinese Herbal Medicine for the Treatment of Vascular Dementia. *2nd International Congress for Complementary Medicine Research*, Munich, May 2007.
45. Jelic V et al. Clinical trials in mild cognitive impairment: lessons for the future. *J. Neurol. Neurosurg. Psychiatry*. 2006; 77:429-438.
46. Jianping Jia, Cuibai Wei, Shuoqi Chen, Fangyu Li, Yi Tang, Wei Qin, Lu Shi, Min Gong, Hui Xu, Fang Li, Jia He, Haiqing Song, Shanshan Yang, Aihong Zhou, Fen Wang, Xiumei Zuo, Changbiao Chu, Junhua Liang, Longfei Jia, Serge Gauthier. (2018). Efficacy and safety of the compound Chinese medicine SaiLuoTong in vascular dementia: A randomized clinical trial. Alzheimer's & Dementia: Translational Research & Clinical Interventions, 4, 108-117. https://doi.org/10.1016/j.trci.2018.02.004.
47. Faul, F., Erdfelder, E., Lang, A.-G., & Buchner, A. (2007). G*Power 3: A flexible statistical power analysis program for the social, behavioral, and biomedical sciences. Behavior Research Methods,39, 175-191.
48. Roman GC, et al. Vascular dementia: diagnostic criteria for research studies. Report of the NINDS-AIREN International Work Group, *Neurology* 1993; 43:250-260.
49. Council for International Organisations of Medical Sciences (CIOMS) in collaberation with World Health Organisation (WHO). *International Ethical Guidelines for Biomedical Research Involving Human Subjects*. Geneva: CIOMS, 2002.
50. National Health and Medical Research Council (NHMRC). National Statement on Ethical Conduct in Research Involving Humans. Canberra, 1999.
51. International Conference on Harmonisation of Technical Requirements for Registration of Pharmaceuticals for Human Use: ICH Harmonised Tripartite Guideline; 1994. http://www.ifpma.org/ich5e.html

52. Dedov VN, Chang D,Liu JX,Sucher NJ. Effect of Wei Nao Kang (*SLT*), a herbal formula for vascular dementia, on the activity of cytochrome P450 isoenzymes. *8th Meeting of Consortium for Globalization of Chinese Medicine (CGCM)*, Nottingham, UK, August 2009.
53. Mohs R. ADAS-cog: what, why and how? 2004:http://alzheimer-insights.com/insights/vol3nol/vol3nol.htm
54. Ferris S.H. General Measures of cognition. International Psychogeriatrics;15(Suppl 1):215-217.
55. Galasko D et al. An inventory to assess activities of daily living for clinical trials in Alzheimer's disease: the Alzheimer's disease Cooperative Study. *Alzheimer Dis Assoc Dis*1997; 11:Suppl 2:S33-S39.
56. Schneider, L. S., Olin, J. T., Doody, R. S., Clark, C. M., Morris, J. C., Reisberg, B., et al. (1997). Validity and reliability of the Alzheimer's Disease Cooperative Study-Clinical Global Impression of Change. The Alzheimer's Disease Cooperative Study. Alzheimer Disease and Associated Disorders, 11 Suppl 2, S22-32.
57. Sellal, F., Wolff, V., & Marescaux, C. The cognitive pattern of vascular dementia and its assessment. *Seminars in Cerebrovascular Diseases and Stroke*. 2004;4:79-86.
58. Royall, D.R., Cordes, J.A., & Polk, M. CLOX an executive clock dawing task. *Journal of Neurology Neurosurgery Psychiatry*. 1998; 64:588-594.
59. Royall, D.R., Mahurin, R.K., & Gray, K.F. Beside assessment of executive cognitive impairment: the executive interview. *Journal of the American Geriatric Society*. 1992; 40:1221-1226.
60. Royall, D.R., Rauch, R., Roman, G.C., Cordes, J.A., & Polk, M.J. Frontal MRI findings associated with impairment on the executive interview (EXIT25). *Experimental Aging Research*. 2001; 27:293-308.
61. Ballard, C., Neil, D., O'Brien J., McKeith I.G., Ince, P. & Perry, R. Anxiety, depression and psychosis in vascular dementia: prevalence and associations. *Journal of Affective Disorders*. 2000; 59(2):97-106.
62. Smith, S.C., Lamping, D.L., Banerjee, S., et al. Measurement of health-related quality of life for people with dementia: development of a new instrument (DEMQOL) and an evaluation of current methodology. Health Technology Assessment 2005; 9(10):1-93.
63. Diamond, E.L., Miller, S., Dickerson, B.C., et al. Relationship of fMRI activation to clinical trial memory measures in Alzheimer disease. *Neurology*2007; 69: 1331-1341.
64. Cummings, J.L., Mega, M.S., Gray, K., Rosemberg-Thompson, S., Gornbein, T. The Neuropsychiatric Inventory: Comprehensive assessment of psychopathology in dementia. *Neurology* 1994; 44:2308-2314.
65. de Medeiros, K., Robert, P., Gauthier, S., et al.. The Neuropsychiatric Inventory-Clinician rating scale (NPI-C): reliability and validity of a revised assessment of neuropsychiatric symptoms in dementia. Int. Psychogeriatr. 2010; 22(6):984-94.
66. Wen, W., & Sachdev, P. S. (2004). Extent and distribution of white matter hyperintensities in stroke patients: the Sydney Stroke Study. Stroke, 35(12), 2813-2819.
67. Wen, W., Sachdev, P.S., Li, J.J., Chen, X., Anstey, K.J., 2009. White matter hyperintensities in the forties: their prevalence and topography in an epidemiological sample aged 44-48. Hum Brain Mapp 30, 1155-1167.
68. http://www.mccauslandcenter.sc.edu/CRNL/tools/pwi

## APPENDIX 1

Adverse Event Definitions

The following definitions are from International Harmonised Tripartite Guidelines and used as the TGA clinical trial guidelines.

Adverse Event

An Adverse Event is any untoward medical occurrence in a patient or clinical investigation subject administered a pharmaceutical product and which does not necessarily have a causal relationship with the treatment. An Adverse Event can therefore be any unfavourable and unintended sign, symptom or disease temporally associated with the use of a medicinal (investigational) product, whether or not related to the medicinal product.

Severity of Adverse Event

Mild Adverse Event: The event causes minimal discomfort and does not significantly interfere with the patient's normal activities.

Moderate Adverse Event: The event is sufficiently uncomfortable to cause some impairment to the participant's normal activities.

Severe Adverse Event: The event is incapacitating and prevents the participant from participating in normal activities.

Serious Adverse Event

A Serious Adverse Event is any untoward medical occurrence that at any dose:

1) Results in death

2) Is life threatening (at immediate risk of death at the time of the event and does not mean an event which hypothetically might have caused death if it was more severe of caused permanent or significant disability / incapacity)

3) Requires inpatient hospitalisation or prolongation of existing hospitalisation

4) Results in persistent or significant disability/ incapacity

5) Leads to a congenital anomaly or birth defect

6) Leads to events that require medical or surgical intervention to prevent permanent impairment of function or damage to body structure

Severe refers to the severity of the event at the time as opposed to the seriousness of the participant's event outcome e.g. a headache might be severe and disabling but not serious. A mild heart attack though not disabling at the time has more serious health consequences.

Causal Event Definitions for Serious Adverse Event Reporting

A Serious Adverse Event (SAE) will be reported in accordance with NHMRC and TGA guidelines. For this purpose, the causal event must also be classified as follows:

1) Causal event is highly probable if a time relationship exists, no other causative factor(s) exists, there is improvement on dose reduction or dechallenge and reoccurrence on rechallenge has occurred (if performed) or if a specific laboratory test has confirmed the relationship.

2) Causal event is probable if a time relationship exists, the event is more likely explained by the study drug than other cause or there is improvement on dechallenge or dose reduction.

3) Causal event is possible if a time relationship exists but other possible causative factor may exist. There may or may not be improvement on dechallenge or dose reduction.

4) Causal relationship is unlikely if time relationship is non-existent or doubtful and /or other factor(s) are certain or probable to have been causative. Causal relationship in cases where the disease under study has deteriorated due to lack of effect should be classified as unlikely.

5) Causal relationship is not related if a time relationship is non-existent or the cause of the event had been identified or the drug cannot be implicated.

# APPENDIX 2

Key Trial Contact Details

| Name | Contact Details | Office |
|---|---|---|
| Prof Dennis Chang | NICM Health Research Institute (NICM), Western Sydney University<br>Locked Bag 1797, NSW 2751 Australia<br>Tel: (+61 2) 4620 3920<br>Fax: (+61 2) 9685 4760<br>E-mail: D.Chang@westernsydney.edu.au | NICM, Building J, Westmead Campus Western Sydney University, NSW |
| Dr Diana Karamacoska | NICM Health Research Institute (NICM), Western Sydney University<br>Locked Bag 1797, NSW 2751 Australia<br>Mob: +61 479 150 816<br>Tel: (+61 2) 9685 4723<br>Fax: (+61 2) 9685 4760<br>E-mail: d.karamacoska@westernsydney.edu.au | NICM, Building J, Westmead Campus Western Sydney University, NSW |
| Sharon Sampath | NICM Health Research Institute (NICM), Western Sydney University<br>Locked Bag 1797, NSW 2751 Australia<br>Mob: +61 412 239 040<br>Fax: (+61 2) 9685 4760<br>E-mail: s.sampath@westernsydney.edu.au | NICM, Building J, Westmead Campus Western Sydney University, NSW |
| Prof Alan Bensoussan | NICM Health Research Institute (NICM), Western Sydney University<br>Locked Bag 1797, NSW 2751 Australia<br>Tel:(+61 2) 4620 3709<br>Fax: (+61 2) 9685 4760<br>E-mail: a.bensoussan@westernsydney.edu.au | NICM, Building J, Westmead Campus Western Sydney University, NSW |
| Prof Henry Brodaty | Dementia Collaborative Research Centre<br>Tel: (+61 02) 9385 2585<br>E-mail: h.brodaty@unsw.edu.au | Dementia Collaborative Research Centre, University of New South Wales. NSW |
| Prof Daniel Chan | Department of Aged Care and Rehabilitation, Bankstown Hospital<br>Tel: (+61 2) 9772 7556<br>E-mail: chan.dky@gmail. com | Department of Aged Care and Rehabilitation, Bankstown Hospital, NSW |

| | | |
|---|---|---|
| Prof Susan Kurrle | Division of Rehabilitation & Aged Care<br>Tel: (+61 2) 9477 9245<br>Fax: (+61 2) 9477 9162<br>Email: susan.kurrle@health.nsw.gov.au | Division of Rehabilitation & Aged Care, Hornsby Ku-ring-gai Hospital, NSW |
| Prof Perminder Sachdev | NPI, Euroa Centre, Prince of Wales Hospital<br>Barker Street<br>Randwick NSW 2031<br>Tel: 02 9382 3763<br>Fax: 9382 3774<br>Email: Perminda.sachdev@unsw.edu.au | Centre for Healthy Brain Ageing (CHeBA). School of Psychiatry, UNSW Medicine, UNSW Australia, NSW |
| Dr Wei Wen | NPI, Euroa Centre<br>Prince of Wales Hospital<br>Barker Street<br>Randwick NSW 2031<br><br>Tel: 02 9382 3730<br>Fax: 02 9382 3774<br>Email: w.wen@unsw.edu.au | Diagnostic MRI Services (DMS), at Neuroscience Research Australia, corner Barker and Easy Streets, Randwick, Sydney NSW |
| Prof Jan Potter | Level 1, Lawson House Wollongong Hospital<br>LMB 8808, South Coast Mail Centre, NSW 2521<br><br>Tel: 02 4222 5035<br>Fax: 02 4222 5764<br>Email: jan.potter@sesiahs.health.nsw.gov.au | Level 1, Lawson House Wollongong Hospital<br>LMB 8808, South Coast Mail Centre, NSW 2521 |
| Dr Dennis<br>Cordato | Clinic A, Level 1, Dept. Neurophysiology, Liverpool Hospital Corner of Elizabeth and Goulburn Streets, Sydney New South Wales 2170<br><br>Tel: 02 8738 3646<br>Fax: 02 8738 3648<br>Email: dennis.cordato@sswahs.nsw.gov.au | Clinic A, Level 1, Dept. Neurophysiology, Liverpool Hospital NSW |
| Dr Mary Ann<br>Kulh | More Than Medicine<br>6/7 53 Mort Street<br>Braddon ACT 2612<br><br>Tel: 02 6162 1639<br>Fax: 02 6162 3202<br>E-mail: makulh@gmail.com | 6/7 53 Mort Street<br>Braddon ACT 2612 |

| | | |
|---|---|---|
| Dr Kannan<br>Natarajan | Internal Medicine & Dementia Research Unit<br>The Prince Charles Hospital<br>4th Floor, Administration Building<br>Rode Road<br>Chermside Qld 4032<br><br>Tel: 07 3139 4000<br>Fax: 07 3139 4923<br>E-mail: Kannan.Natarajan@health.qld.gov.au | Internal Medicine & Dementia Research Unit<br>The Prince Charles Hospital QLD |
| Dr Nawaf Yassi | Department of Neurology, 4 Centre,<br>Royal Melbourne Hospital, Grattan Street<br>Parkville VIC 3050<br><br>Tel: 03 9342 4420<br>Fax: 03 9342 8628<br>E-mail: nawaf.yassi@unimelb.edu.au | Department of Neurology, Royal Melbourne Hospital VIC |
| Dr Kujan Nagaratnam | Suite G12, Norwest Private Hospital, 9 Norbrik Ave, Bella Vista, NSW 2153<br><br>Tel: 0421591640<br>Fax: 02 88835237<br>E-mail: kujan.nagaratnam@gmail.com | Specialist Services Medical Group, Norwest Private Hospital NSW |
| Prof Stephen Myers | NatMed-Research Unit, Division of Research Southern Cross University<br>PO Box 157 Lismore NSW 2480<br><br>Tel: 0421612713<br>E-mail: stephen.myers@scu.edu.au | NatMed-Research Unit, Division of Research Southern Cross University NSW |
| Dr Iveta<br>Valachova | Aged Private Care<br>36 Evans Street<br>Wollongong 2500<br><br>Tel: 0435 511 875<br>Fax: 02 4244 4870<br>E-mail: agedprivatecare@gmail.com | 36 Evans Street<br>Wollongong NSW |

| | | |
|---|---|---|
| Dr Cathy Short | CALHN Memory Trials (RAH and TQEH)<br>Royal Adelaide Hospital<br>Port Rd, Adelaide SA 5000<br><br>Tel: 08 707 43284<br>Fax: 08 8429 6085<br>E-mail: cathy.short@sa.gov.au | CALHN Memory Trials (RAH and TQEH)<br>Royal Adelaide Hospital SA |
| Dr Dennis<br>Cordato | Southern Neurology<br>Suite 2, 19 Kensington St<br>Kogarah NSW 2217<br><br>T: 02 8566 1500<br>F: 02 9553 7090<br>Email: dennis.cordato@sswahs.nsw.gov.au | Southern Neurology<br>Suite 2, 19 Kensington St<br>Kogarah NSW 2217 |

## APPENDIX 3

Clinical Frailty Scale

The Clinical Fraility Scale can be retrieved from:

https://www.dal.ca/sites/gmr/our-tools/clinical-frailty-scale.html

## APPENDIX 4

### SLT001 Trial Contingency Measures

In response to the COVID-19 outbreak in Australia, study procedures have been amended to protect the safety and wellbeing of participants, their caregivers as well as the research team, and to minimise the impact of the outbreak on the SLT001 trial. Recruitment is to be paused until the trial centre's public health directive and their governance body allow for recruitment to resume. All active participants and research staff will be managed to prioritise their safety, and these strategies are detailed in this appendix.

**1. SLT001 Trial Contingency Measures for New Participants**

| | Activity | Pre-COVID Status | Alternate Arrangements | Responsibility | Note/Action |
|---|---|---|---|---|---|
| 1.0 | New Recruitment | F2F screening on-site | Recruitment is currently suspended.<br><br>However, recruitment can recommence at centres where the public health directive and their governance body allow for it.<br>• COVID-19 safety checks and precautions need to be adhered to until the COVID-19 completely resolves when face-to-face assessments are conducted (see Items 1.1 and 1.2 for more details)<br>• For follow-up visits of the new participants, follow the alternate arrangements outlined in Items 2, 3 and 4 according to the situations and requirements of the relevant site. | All Centres | The screening visit must be conducted face-to-face according to current protocol requirements.<br><br>Actions:<br>(1) Centres to check the local public health directive and their governance body for current advice on recruitment and face-to-face appointments and inform the Local Sponsor of this.<br>(2) All centres to keep their potential eligible participants informed of the development and to provide a copy of PIS;<br>(3) Participants will be invited to participate once recruitment resumes |

| | Activity | Pre-COVID Status | Alternate Arrangements | Responsibility | Note/Action |
|---|---|---|---|---|---|
| 1.1 | COVID-19 safety check prior to screening appointment | Not in the current protocol | (1) Screen all potential participants and carers over the phone for cold and flu symptoms, recent overseas or interstate travel and any close contact with a confirmed or suspected COVID-19 case in the past 14-days<br>– Individuals with these symptoms should be referred to qualified facilities for further investigation<br>– Individuals with these symptoms requesting advice should be directed to the Health Direct Line 1800 020 080 or website https://www.healthdirect.gov.au/coronavirus | All Centres | (1) Personnel to conduct COVID-19 safety check with every call to a participant and delegated Investigators must review any reports of symptoms;<br><br>(2) Do not see or assess participant on study instruments, or proceed with pathology test, if symptoms present and cause is being investigated. Delay screening appointment until they have been cleared. |
| 1.2 | Screening visit conduct | Conducted face-to-face on site | The options include:<br><br>1) Visiting the trial centre for these assessments where such visits are permitted; COVID-19 safety check and protection measures must be taken<br><br>2) Verbally consenting to being assessed at a nominated community centre; COVID-19 safety check and protection measures must be taken | All centres | The assessors, participant and caregiver will thoroughly wash their hands and/or use hand sanitizer prior to and following a face-to-face visit. Masks and/or gloves to be used in line with local health advice. Centres will need to ensure PPE supplies are readily accessible if conducting face-to-face visits. |

2. **SLT001 Trial Contingency Measures for Week 4, 13, and 39 Visits**

| . | Activity | Pre-COVID Status | Alternate Arrangements | Responsibility | Note/Action |
|---|---|---|---|---|---|
| 2.0 | Week 4, 13, and 39 visits (non-assessment visits) | Face-to-face on site recording of vital signs, adverse events, safety bloods and dispensing IP in Week 13 | These visits can be conducted at the trial centre or nominated community centre, through a home visit, or remotely as per Items 2.1–2.5<br>• Research personnel must screen for COVID-19 symptoms and refer any suspicious case to qualified facilities for further investigation as per Item 2.3<br>• COVID-19 safety protection measures must be taken with any face-to-face visits | All Centres | Items to follow up: vital signs, adverse events and safety pathology tests.<br><br>Centres will need to assess their current policies and procedures to determine appropriate action for these visits.<br><br>Researchers to obtain verbal consent to a home visit, or to collect this information over the phone, and record this in the source visit notes.<br><br>For face-to-face visits: the assessors, participant and caregiver will thoroughly wash their hands and/or use hand sanitizer prior to and following a face-to-face visit. Masks and/or gloves to be used in line with local health advice. Centres will need to ensure PPE supplies are readily accessible if conducting face-to-face visits. |

| . | Activity | Pre-COVID Status | Alternate Arrangements | Responsibility | Note/Action |
|---|---|---|---|---|---|
| 2.1 | Vital signs (HR, BP, Ht and Wt) | Recorded during the site visit | If unable to do a face-to-face assessment, at the centre or via a home visit, collect this data remotely:<br><br>• Wt to be provided by the participant/caregiver over the phone if they have scales at home, otherwise Wt value to be carried forward from previous reading<br>• Ht to be carried forward from the previous reading<br>• HR and BP to be provided, if possible, by (1) the participant/caregiver if they have a BP facility at home or (2) the study team member or the mobile venipuncturist conducting a home visit. In the latter case, consent needs to be obtained for the team member or venipuncturist to attend home for blood collection and vital sign measurement | All Centres | Document verbal consent to home visit or remote collection of data, and if opting for mobile venipuncturist, in the source visit notes.<br><br>If unable to collect data, use last value carried forward method to address missing data. |
| 2.2 | Adverse events | Recorded during the site visit | Adverse events can be identified and reported over the phone where a face-to-face visit is not feasible | All Centres | Delegated Investigators need to assess the causality of the reported AEs and SAEs, and to follow up with the participant when needed. |

| . | Activity | Pre-COVID Status | Alternate Arrangements | Responsibility | Note/Action |
|---|---|---|---|---|---|
| 2.3 | COVID-19 safety check | Not applicable | (1) Screen all participants over the phone for cold and flu symptoms, recent overseas or interstate travel and any close contact with a confirmed or suspected COVID-19 case in the past 14-days<br>– Participants with these symptoms should be referred to qualified facilities for further investigation<br>– Participants with these symptoms requesting advice should be directed to the Health Direct Line 1800 020 080 or website: https://www.healthdirect.gov.au/coronavirus<br>(2) If the participant is diagnosed with a coronavirus infection, trial medication may be suspended until symptoms resolve | All Centres | (1) Personnel to conduct COVID-19 safety check with every call to a participant and delegated Investigators must review any reports of symptoms.<br><br>(2) Do not proceed with pathology test (Item 2.5) if COVID-19 symptoms present and cause is being investigated. Delay appointment until they have been cleared. We will allow deviations in the visit schedule during this time. |
| 2.4 | IP dispensing (Week 13 only) | A 3-month supply of IP is dispensed on site by delegated personnel | If unable to attend trial centre:<br><br>A 6-month supply of IP can be delivered by a courier service or through a home visit by a study team member, with visit 5 and visit 6 diaries, and instructions to keep empty packaging/leftover capsules | All Centres and relevant hospital pharmacy | Obtain verbal consent to receive 6-month IP supply and record this in source notes. Centres will organise courier to deliver IP. Compliance check: Participants will be asked to keep boxes and return them together with unfinished capsules at next visit |

| . | Activity | Pre-COVID Status | Alternate Arrangements | Responsibility | Note/Action |
|---|---|---|---|---|---|
| 2.5 | 2.5.1 Safety Pathology Testing at Weeks 4 and 13 | Conducted at hospital/local pathology laboratories | If participant passes the COVID-19 checks, participant will be offered to verbally consent to the following two options:<br><br>(1) Continue to use the current pathology lab for the testing when on-site visit to pathology lab is permitted<br><br>(2) Home visit of a qualified study team member or mobile venipuncturist to take blood and deliver to the pathology lab for analysis; infection control procedures will be enacted as per Item 3.1<br><br>If the participant declines to take either option, the medical history and previous pathology results of the participant will be assessed by the PI in consultation with the coordinating CI to determine if this participant can continue in the trial | All Centres | For Option 1, Participant will be provided with a pathology slip; Sponsor to cover the costs of taxi/Uber if needed to avoid public transport.<br><br>For Option 2, Sponsor to cover the mobile blood collection services if relevant.<br><br>Centres to record verbal consent to remote blood collection in source visit notes. |
| | 2.5.2 Safety Pathology Testing at Week 39 | Conducted at hospital/local pathology laboratories | To further minimise risk, the Week 39 pathology test can be skipped if there are no abnormal and unresolved findings at Week 26. Otherwise follow 2.5.1 for pathology testing. | All Centres | If abnormalities are recorded in the previous tests, Week 39 pathology test will be conducted as per Item 2.5.1 |

## 3. SLT001 Trial Contingency Measures for Weeks 0, 26 and 52 Visits

| . | Activity | Pre-COVID Status | Alternate Arrangements | Responsibility | Note/Action |
|---|---|---|---|---|---|
| 3.0 | Week 0, 26 and 52 visits (assessment visits) | Face-to-face on-site | Part of the testing including VaDAS-cog, Exit-25, CLOX, CIBIS/CIBIC plus will be conducted as per Item 3.1.<br><br>Part of the testing including ADCS-ADL, DEMQOL, DEMQOL-Carer and NPI can be conducted remotely (i.e., over the phone) to reduce face-to-face contact time as per Item 3.2 | All Centres | Centres will need to assess their current policies and procedures to determine appropriate action for these visits.<br><br>Researchers to obtain verbal consent to collect any data remotely and record this in the source visit notes. |
| 3.1 | VaDAS-cog, Exit-25, CLOX, CIBIS/CIBIC plus | Conducted face-to-face on site | The options include:<br><br>1) Verbally consenting to continuing to visit the trial centre for these assessments at the site where such visits are still permitted; COVID-19 safety check and protection measures must be taken as per Item 3.4<br><br>2) Verbally consenting to being assessed at a nominated community centre; COVID-19 safety check and protection measures must be taken as per Item 3.4<br><br>3) Verbally consenting to receive a home visit from assessors; COVID-19 safety check and protection measures must be taken as per Item 3.4 | All centres | For Options 1 & 2, Sponsor to cover the costs of taxi/Uber if needed to avoid public transport.<br><br>For face-to-face visits: the assessors, participant and caregiver will thoroughly wash their hands and/or use hand sanitizer prior to and following a face-to-face visit. Masks and/or gloves to be used in line with local health advice. Centres will need to ensure PPE supplies are readily accessible if conducting face-to-face visits.<br><br>If unable to collect data, record as Not Done and explain why in the source notes. |

| | | | | | |
|---|---|---|---|---|---|
| | | | 4) For the sites where both on site and home visits are not feasible, and/or participants are unwilling to attend in person, the possibility of using telehealth or videoconferencing to conduct these assessments will be explored; this option is not viable for the baseline visit (Week 0) | | |
| . | Activity | Pre-COVID Status | Alternate Arrangements | Responsibility | Note/Action |
| 3.2 | ADCS-ADL, DEMQOL, DEMQOL-carer, and NPI | Conducted face-to-face on site | These tests may be conducted remotely via phone to reduce face-to-face assessment time of Item 3.1, Options 1, 2, and 3. | All Centres | Researchers to obtain verbal consent to collect any data over the phone and record this in the source visit notes. |
| 3.3 | IP dispensing (Week 26 only) | Dispensed on site by the hospital pharmacy or investigator | Two available options:<br><br>(1) Centres where no on-site visits are possible: 6-month supply of IP to be delivered by a courier service or through a home visit by a study team member, with visit 6 and 7 diaries, and instructions to keep empty packaging/leftover capsules<br><br>(2) Centres where site visits are still allowed: IP to be collected from pharmacy or delegated personnel | All Centres | Obtain verbal consent to receive 6-month IP supply and record this in source notes.<br><br>Centres will organise delivery of IP.<br><br>Compliance check: Participants will be asked to keep boxes and return them together with unfinished capsules at next visit. |

| . | Activity | Pre-COVID Status | Alternate Arrangements | Responsibility | Note/Action |
|---|---|---|---|---|---|
| 3.4 | COVID-19 safety check | Not applicable | (1) Screen all participants over the phone for cold and flu symptoms, recent overseas or interstate travel and any close contact with a confirmed or suspected COVID-19 case in the past 14-days<br>– Participants with these symptoms should be referred to qualified facilities for further investigation<br>– Participants with these symptoms requesting advice should be directed to the Health Direct Line 1800 020 080 or website https://www.healthdirect.gov.au/coronavirus<br>(2) If the participant is diagnosed with a coronavirus infection, trial medication may be suspended until symptoms resolve | All Centres | (1) Personnel to conduct COVID-19 safety check with every call to a participant and delegated Investigators must review any reports of symptoms;<br><br>(2) Do not see or assess participant on study instruments, or proceed with pathology test, if symptoms present and cause is being investigated. Delay appointment until they have been cleared. |
| 3.5 | Safety Pathology Testing | Conducted at hospital/local pathology laboratories | If participant passes the COVID-19 checks, participant will be offered to verbally consent to the following two options:<br>(1) Continue to use the current pathology lab for the testing when on-site visit to pathology lab is permitted<br>(2) Home visit of a study team member or a mobile venipuncturist to take blood and deliver to the pathology lab for analysis; infection control procedures will be enacted as per Item 3.1<br><br>If the participant declines to take either option, the medical history and previous pathology results of the participant will be assessed by the PI and coordinating CI to determine if this participant can continue in the trial | All Centres<br><br>NICM | For Option 1, Pathology slip will be provided to participant; Sponsor to cover the costs of taxi/Uber if needed to avoid public transport.<br><br>For Option 2, Sponsor to cover the mobile blood collection services if relevant.<br><br>Centres to record verbal consent to remote blood collection in source visit notes. |

4. **SLT001 Trial Contingency Measures for Week 65 Visit**

| . | Activity | Pre-COVID Status | Alternate Arrangements | Responsibility | Note/Action |
|---|---|---|---|---|---|
| 4.0 | Week 65 visits (assessment visits) | Face-to-face on-site assessment for safety, VADAS-Cog, ADLs, and CIBIC | The options include:<br><br>1) Verbally consenting to continuing to visit the trial centre for these assessments at the site where such visits are still permitted; COVID-19 safety check and protection measures must be taken as per Item 3.4<br><br>2) Verbally consenting to being assessed at a nominated community centre; COVID-19 safety check and protection measures must be taken as per Item 3.4<br><br>3) Verbally consenting to receive a home visit from assessors; COVID-19 safety check and protection measures must be taken as per Item 3.4<br><br>4) For the sites where both on site and home visits are not feasible, and/or participants are unwilling to attend in person, the possibility of using telehealth or videoconferencing to conduct these assessments will be explored | All Centres | Centres will need to assess their current policies and procedures to determine appropriate action.<br><br>Researchers to obtain verbal consent to collect any data remotely and record this in the source visit notes.<br><br>For face-to-face visits: the assessors, participant and caregiver will thoroughly wash their hands and/or use hand sanitizer prior to and following a face-to-face visit. Masks and/or gloves to be used in line with local health advice. Centres will need to ensure PPE supplies are readily accessible if conducting face-to-face visits.<br><br>If unable to collect data, record as Not Done and explain why in the source notes. |


| . | Activity | Pre-COVID Status | Alternate Arrangements | Responsibility | Note/Action |
|---|---|---|---|---|---|

| | | | | | |
|---|---|---|---|---|---|
| 4.1 | Post-trial supply of 12 month open-label medication | Dispensed on site by the hospital pharmacy or investigator | If face-to-face visit not feasible, and participant and Investigator proceed with 12 month open-label supply of SLT medication, researchers must obtain verbal consent to deliver IP with post-trial adverse event diary. This can be done through a courier service, or by a study team member conducting a home visit. | NICM and All Centres | Researchers to obtain verbal consent to have post-trial IP and visit diary delivered, and record this process in source visit notes; NICM will continue the remote monitoring of their safety via regular phone calls. |
| 4.2 | Post-trial Safety Pathology Testing | Conducted at hospital/local pathology laboratories | Laboratory studies will be conducted only if an adverse event is reported in the post-trial period and the Investigator deems it necessary to perform this test; These will be carried out in accordance with Item 3.5. | All Centres | Refer to Item 3.5 for the conduct of Investigator initiated laboratory studies. |

## 5. SLT001 Trial Other Contingency Measures

| . | Activity | Pre-COVID Status | Alternate Arrangements | Responsibility | Note/Action |
|---|---|---|---|---|---|
| 5.1 | Back-ups to CIs, PIs, coordinators and raters | N/A | All centres and central team will be required to nominate a back-up person for each of their key team members who will be deputised if he/she is unwell | NICM and All Centres | All centres to prepare a list of back-ups for their team and email this to the trial coordinator. This includes ensuring access to relevant hard copy and electronic files.<br><br>The alternate contact list for the NICM team is provided on the next page. |
| 5.2 | Visit window | The current visit window is ± 7 days | Visit window is extended to ± 28 days during the COVID-19 pandemic for all visits | NICM | |
| 5.3 | Letter to participant | N/A | A letter will be sent to all active participants and their caregivers to inform them of changes to our trial conduct to encourage them to stay in the trial during the pandemic | NICM and All Centres | NICM will liaise with the centres to prepare and deliver this letter. |
| 5.4 | Follow-up phone call | N/A | A follow-up phone call from site coordinators to each of their participants and caregivers to discuss questions and issues | NICM and All Centres | Centres to document the issuing of letter from Item 5.3 and follow-up call in source notes. |

**Alternate Contacts for NICM Central Team Members**

These are the contact details for the NICM central coordinating team and their nominated back-ups. These nominated individuals will deputise when a team member becomes unwell. Should this occur, out-of-office emails will be activated that contain the nominated individual's contact details.

| Team Member | Email | Phone | Nominated Contact(s) |
|---|---|---|---|
| Dennis Chang | d.chang@westernsydney.edu.au | +61 404 453 682 | Alan/Diana |
| Alan Bensoussan | a.bensoussan@westernsydney.edu.au | +61 404 055 292 | Dennis |
| Diana Karamacoska | d.karamacoska@westernsydney.edu.au | +61 479 150 816 | Dennis/Lena |
| Lena Hattom | l.hattom@westernsydney.edu.au | +61 421 323 088 | Diana/Dennis |
| Suzannah Bourchier (Mon – Wed) | s.bourchier@westernsydney.edu.au | +61 419 600 119 | Dennis/Diana |

## COVID-19 Decision Making Flow Chart

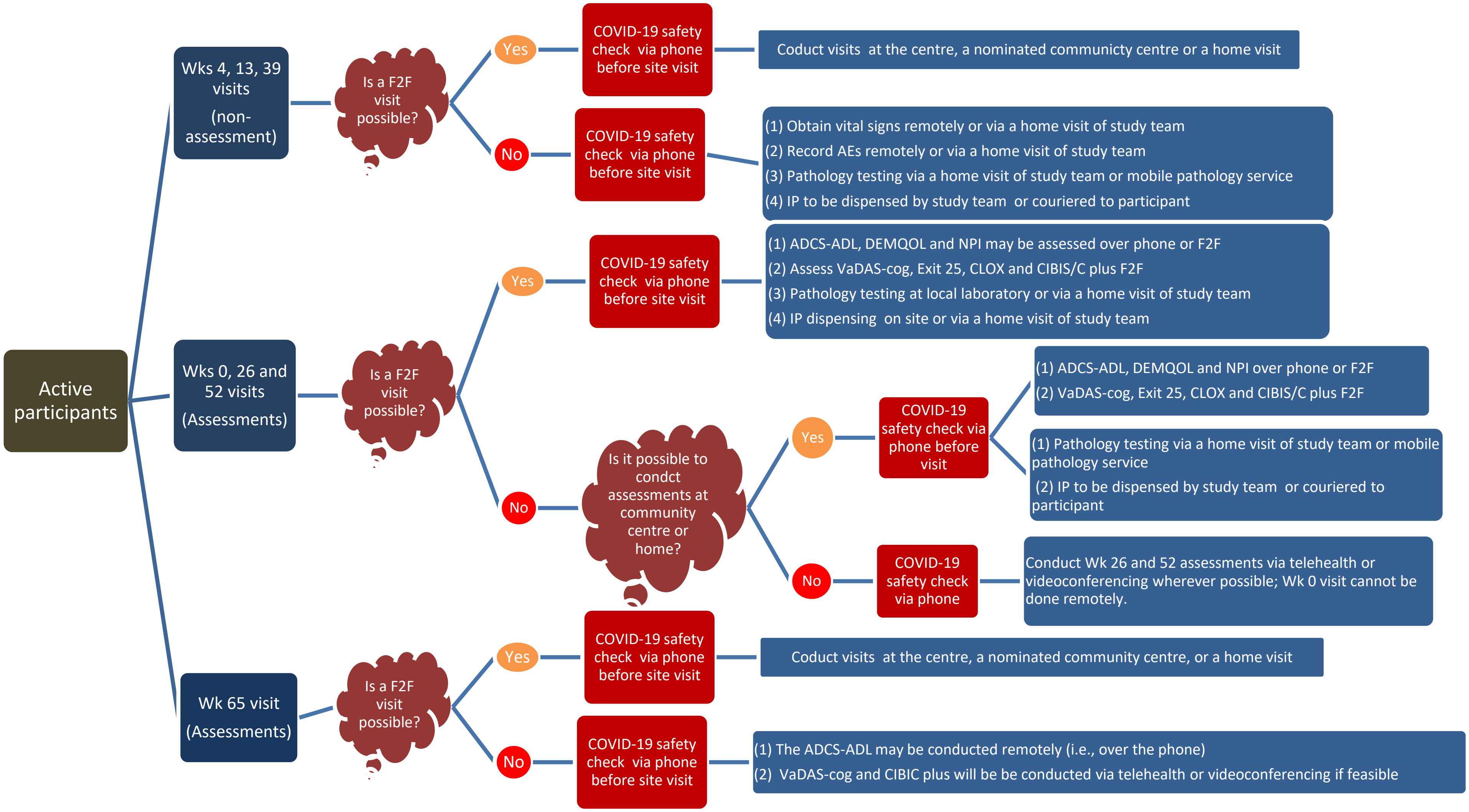

Supplement: S1 File — (PDF) [file pone.0265285.s002.pdf]
